# Progress and Application of Halide Perovskite Materials for Solar Cells and Light Emitting Devices

**DOI:** 10.3390/nano14050391

**Published:** 2024-02-20

**Authors:** Maoding Cheng, Jingtian Jiang, Chao Yan, Yuankun Lin, Mansour Mortazavi, Anupama B. Kaul, Qinglong Jiang

**Affiliations:** 1 School of Material Science and Engineering, Jiangsu University of Science and Technology, Zhenjiang 212100, Chinachaoyan@just.edu.cn (C.Y.); 2 Department of Chemistry and Physics, University of Arkansas at Pine Bluff, Pine Bluff, AR 71601, USA; 3 Department of Physics, University of North Texas, Denton, TX 76203, USA; yuankun.lin@unt.edu; 4 Department of Electrical Engineering, University of North Texas, Denton, TX 76207, USA

**Keywords:** halide perovskite, solar cells, LED, light emitting, nano, efficiency

## Abstract

Halide perovskite materials have attracted worldwide attention in the photovoltaic area due to the rapid improvement in efficiency, from less than 4% in 2009 to 26.1% in 2023 with only a nanometer lever photo-active layer. Meanwhile, this nova star found applications in many other areas, such as light emitting, sensor, etc. This review started with the fundamentals of physics and chemistry behind the excellent performance of halide perovskite materials for photovoltaic/light emitting and the methods for preparing them. Then, it described the basic principles for solar cells and light emitting devices. It summarized the strategies including nanotechnology to improve the performance and the application of halide perovskite materials in these two areas: from structure–property relation to how each component in the devices affects the overall performance. Moreover, this review listed the challenges for the future applications of halide perovskite materials.

## 1. Introduction 

**Figure 1:**
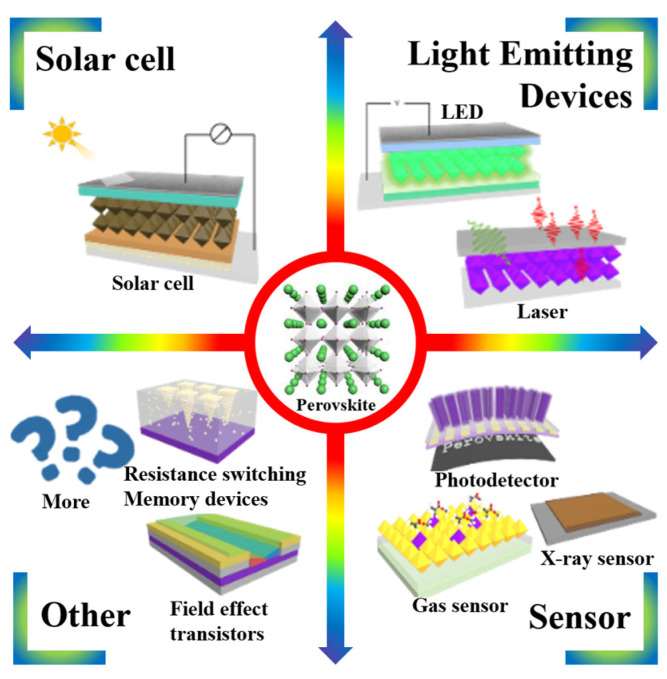
Various applications of halide perovskite materials.

In recent years, halide perovskite materials have attracted strong and wide attention due to the excellent optical and electrical properties, such as a long free carrier diffusion length, high charge carrier mobility, tunable band gap, high photoluminescence quantum yield (PLQY), and solution processability. They usually have the general formula ABX_3_, where A is organic cation such as CH_3_NH_3_^+^(MA), CH(NH_2_)_2_^+^(FA), C_6_H_5_(CH_2_)_2_NH_3_^+^(PEA), or inorganic cation such as Cs^+^, Rb^+^; B is group IV element such as Pb^2+^ or Sn^2+^; X is I^−^, Br^−^, Cl^−^ ion [1,2,3,4,5]. The first report of halide perovskite material in the field of optoelectronics was a halide perovskite sensitized solar cell developed by Miyaska in 2009 [6]. Over a decade of development, the highest PCE in the halide perovskite solar cells has reached 26.1% [7]. This PCE value is already close to the highest value of silicon-based solar cell, which is over a half century in history [8]. In addition to the successful application of halide perovskite materials in solar cells, various other applications expanded the magic of halide perovskite materials, such as light emitting diodes (LEDs) [9,10,11], photodetectors [12,13,14,15,16,17,18,19,20,21,22,23], field effect transistors [24,25,26], gas sensors [27], resistance switching memory devices [28,29,30,31,32], laser and light emitting devices [33], as shown in Figure 1 [34,35]. Among these, halide perovskite materials-based light emitting devices exhibit unprecedented performance with external quantum efficiencies (EQEs) exceeding 28.2% [36]. More interestingly, the light of all visible wavelengths (colors of light) can be achieved simply by changing the halogen anions or the ratio of halogen anions. The high performance of halide perovskite light emitting can be attributed to the inherent properties of halide perovskite materials such as low defect density, high crystallinity, high absorption, high PLQY, and efficient charge transport.

### 1.1. Halide Perovskite Materials

**Figure 2:**
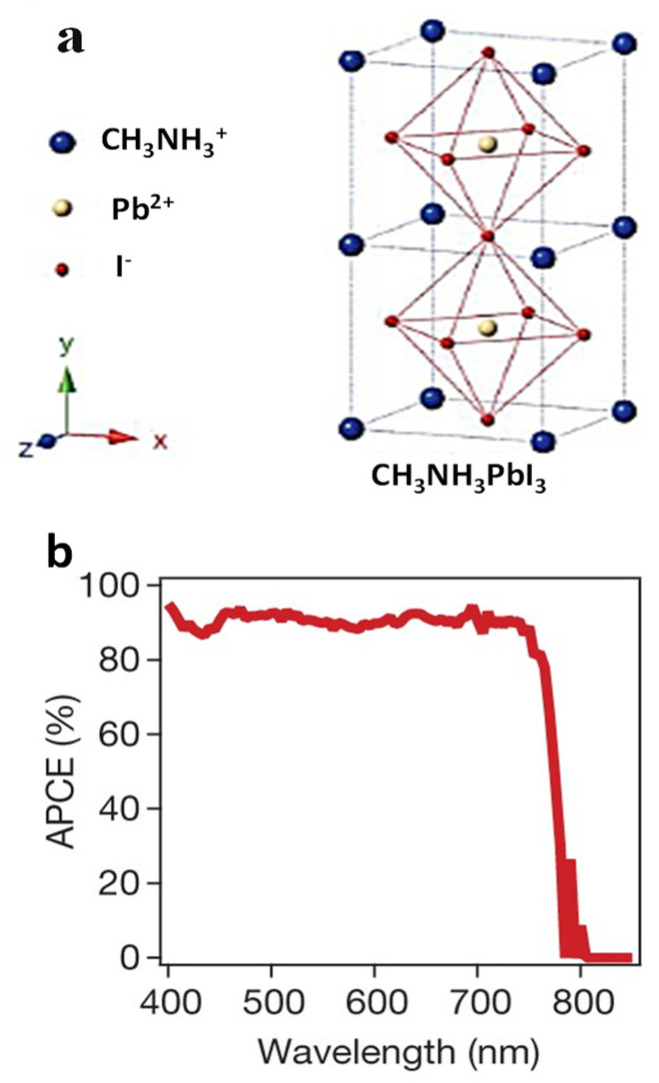
(**a**) FCC (face centred cubic) structure of halide perovskite CH_3_NH_3_PbI_3_ and (**b**) absorbed photon-to-current conversion efficiency (APCE).

In a typical halide perovskite type organic/inorganic hybrid material as shown in Figure 2a, CH_3_NH_3_PbI_3_ (AMX_3_) crystal is an orthorhombic P_nma_ space group. The CB (conduction band) and VB (valence band) are −3.93 eV and −5.43 eV with a band gap of 1.5 eV, which means that halide perovskite can absorb light as long as 800 nm, as shown in Figure 2b. CH_3_NH_3_PbI_3_ can have different crystal symmetries and give both cubic and tetragonal crystals. Goldschmidt’s tolerance factor (t factor) can be used as an empirical parameter to predict the stability and distortion of halide perovskite crystal structures (AMX_3_).

(1) $$t=\left(R_{A}+R_{X}\right)/\sqrt{2}\left(R_{M}+R_{X}\right)$$

*R_A_*, *R_M_*, and *R_X_* are the ionic radii for *A*, *M*, and *X*. When *t* = 1, the crystal structure has the maximum stability and distortion is expected when t deviates from unity. Usually, the cubic halide perovskite crystal formed when 0.9 < *t* < 1 [37]. A large *M* ion or small *A* ion cause the *t* factor to vary between 0.7 and 0.9, resulting in orthorhombic, rhombohedral or tetragonal structures. Ruddlesden–Popper phase (RP, layered halide perovskite) structures were found when *t* is larger than unity [38,39].

### 1.2. Optical Properties of Halide Perovskites

The spatial configuration of an atom and its nature determine the electron valence and optical transition probability in its molecules and crystal lattice. The electrons in the atom will occupy the discontinuous energy state; therefore, it can produce some narrow absorption and emission lines. However, in semiconductors, the electrons in the conduction band and the holes in the valence band are separated by a forbidden band, which produces an absorption and emission spectrum that is completely different from the atomic spectrum.

As the game changer in photovoltaics area, halide perovskite materials exhibit striking excellence performance in light absorption (over 1.5 × 10^4^/cm at 550 nm) [40,41], charge transportation (1069 nm electron diffusion length and 1213 nm holes diffusion length) [41]. In halide perovskite materials, the symmetry and the lone-pair s orbitals enable the direct band gap p-p transition, which usually is much stronger than the p-s transition in the other materials for thin-film solar cells such as Cu(In,Ga)Se_2_(CIGS) and CdTe [42].

**Figure 3:**
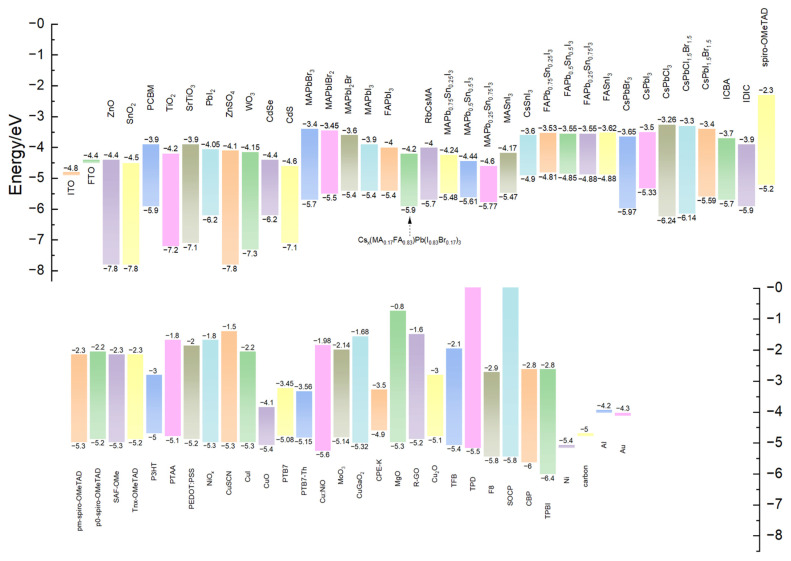
Band gap diagram of halide perovskite materials and transport layer.

For semiconductor materials under irradiation such as halide perovskites, electrons are excited from the VB to the CB, leaving holes in VB. Electrons and holes move freely in CB and VB to form excitons. Moreover, the Bohr radius will extend over several lattice constants in the plane of the inorganic halide perovskite. Thereby, the recombination of electrons and holes produces a strong light emission [43]. The salient feature of this system is that the exciton state has great binding energy and oscillator strength. For example, the exciton binding energy of MAPbBr_3_ is 2.258 eV [44] and the exciton binding energy of MAPbI_3_ is 1.633 eV [45], compared with the exciton binding energy of bulk phase PbI_2_ of only 30 meV. Figure 3 shows the bandgap that embodies the different halide perovskites as well as the transport layer and the metal electrodes. According to Ishihara’s research, organic–inorganic halide perovskites have an organic–inorganic layered structure and a dielectric confinement effect [46]. The lower dielectric constant of inert organic molecules results in reduced screening of carriers in these layers and enhances Coulomb interactions in the process of combining electron–hole pairs to form excitons. Therefore, even at room temperature, this is the reason for achieving a strong photoluminescence of the layered halide perovskite mixture. And halide perovskite materials have excellent band gap tuneable properties. The wavelength of the emitted light can be easily changed by adjusting the type and proportion of the halogens, and the excellent performance of the visible band gap can be adjusted [47] or the different B elements can also change the emission wavelength of the halide perovskite materials [48]. Zhang et al. found that for halide perovskite crystals [49], there was also a red shift of the band gap caused by volume compression under high pressure. Thus, the wavelength (nm) of the emitted light can also be changed.

### 1.3. Electrical Properties for Halide Perovskite Materials

The source of electrical properties of halide perovskite materials has been widely debated. It is reported that the electrical properties of halide perovskite materials are caused by the relative movement of halogen elements [50,51]. However, some studies have reported different views by studying the ASnI_3_ halide perovskite, which is believed to be caused by the transmission of electron charges [52,53,54,55,56]. The cause of the contradiction that causes ASnX_3_ to have both conductivity and semiconducting properties is due to differences in synthetic methods. It exhibits a metal p-type behaviour when synthesized by a high-temperature melting method and it exhibits an n-type intrinsic semiconductor property when used at a low-temperature synthesis [57,58].

However, both ASnX_3_ and APbX_3_ halide perovskites have problems with the stability from the element Sn or Pb. For example, the halide perovskite structure changes and becomes unstable when Sn^2+^ is oxidized to Sn^4+^, which also has a great influence on the performance of the devices. Therefore, different preparation methods can also prepare lead-based halide perovskites with different electrical properties [59,60,61]. As a result, the halide perovskite can form a certain charging defect by continuous phase transformation and utilizing the displacement of the lattice atom during the phase transformation. Due to the charged defect, the electrostatic potential can be generated without changing the stoichiometry of the material, thereby affecting the carrier density. Thus, we can prepare the halide perovskite of the crystal structure and electrical properties based on the requirements.

### 1.4. Synthesis of Halide Perovskite Materials

#### 1.4.1. Solution Method

One-step method. Generally, the salt solution of the precursor dissolves in a solvent such as DMF or DMSO, and then crystallizes by evaporation of the solvent to obtain halide perovskite crystals. Among them, the ion concentration and the evaporation rate have a great influence on the formation of crystals. Bao et al. found that adding different additives to the halide perovskite precursor solution can control the rate of crystallization of halide perovskite [62]. Zhang et al. found that lead acetate can increase the crystallization rate of halide perovskite with solid state crystallization using a non-halide lead source (lead acetate) instead of lead chloride or iodide [63].

**Figure 4:**
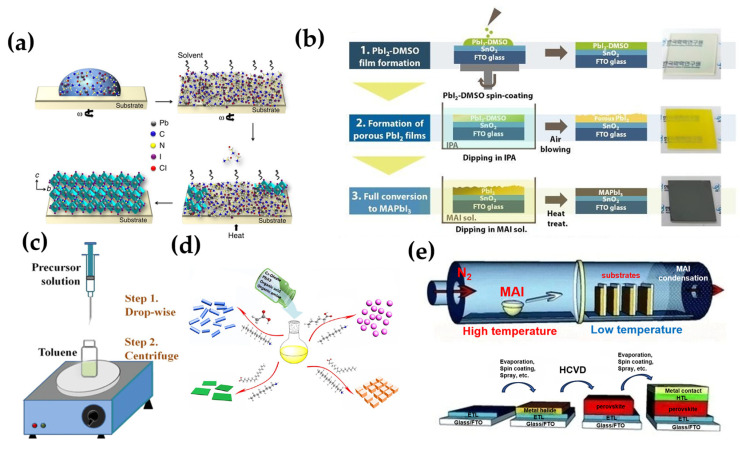
(**a**) During the solution coating process, solvent evaporation occurs but the excess organic component remains within the film, the removal of which by following thermal annealing leads to the fully crystallized perovskite thin film [63]. (**b**) A schematic diagram of MET representing each procedure and optical images of the resulting films [64]. (**c**) Schematic illustration of the reaction system and process for LARP technique [76]. (**d**) Illustration for the formation process of different CsPbX_3_ (X = Cl, Br, I) nanocrystals mediated by organic acid and amine ligands at room temperature. Hexanoic acid and octylamine for spherical quantum dots; oleic acid and dodecylamine for nanocubes; acetate acid and dodecylamine for nanorods; oleic acid and octylamine for few-unit-cell-thick nanoplatelets [77]. (**e**) Diagram of the HCVD furnace and MAI deposition onto metal halide seeded substrates [72].

Two-step method. In the two-step method, a substance such as lead halide having a small solubility is generally dissolved in a polar solvent such as DMSO, and then spin-coated, vapor-deposited or immersed in a substance containing a Cesium halide. Kim et al. pre-coated lead iodide in DMSO, and then excess DMSO was removed to form a film having a porous morphology and an unusual crystal orientation [64]. PbI_2_ was completely converted to MAPbI_3_ by the addition of subsequent MAI (Figure 4b).

#### 1.4.2. Hot Injection Crystallization

The hot injection crystallization method is the primary method for preparing high-brightness halide perovskite materials, and the halide perovskite nanocrystallites are obtained by injecting a halide perovskite precursor at a high temperature and then rapidly cooling the solution to make the solution supersaturated, as shown in Figure 4c. According to a report, Song pioneered the use of hot injection to prepare a halide perovskite quantum dot with an adjustable band gap [47]. Protesescu et al. also successfully prepared a wide-gamut halide perovskite in the visible range by this method [65]. Shamsi also relied on this method to prepare quantum-limited CsPbBr_3_ nanosheets [66]. Moreover, the doping of halide perovskite can be achieved subtly using the hot injection method. Parobek successfully formed the intermediate structure by thermal injection before the thermal injection of the Cs precursor, and successfully synthesized the Mn-doped CsPbBr_3_ nanocrystals. It has been reported that the use of hot injection can also achieve the protection of the package of halide perovskite nanosheets [67]. Zhong et al. used the hot injection method to synthesize CsPbBr_3_@SiO_2_ core-shell nanoparticles, which can maintain long-term stability in water [68].

#### 1.4.3. Anti-Solvent Crystallization

Generally (in Figure 4d), the anti-solvent crystallization method dissolves the halide perovskite precursor in a polar solvent such as DMF and DMSO, and then a non-polar poor solvent was added, such as hexane or toluene. According to a report, Michele dissolved the halide perovskite precursor in dimethyl sulfoxide (DMSO), then added diethyl ether (DE) to act as an anti-solvent, and successfully prepared Cs_4_PbBr_6_ crystal [69]. However, toluene and chloroform as anti-solvents are generally more toxic. Zhang et al. used methoxybenzene as an anti-solvent to successfully prepare a halide perovskite solar cell with an efficiency of up to 19% [70].

#### 1.4.4. Vapor Deposition

In the vapor deposition method shown in Figure 4e, a halide perovskite precursor in solution can be spin-coated on a substrate such as FTO glass, and then methylamine or the similar solvent is vaporized at the other end to react with a halide perovskite precursor on the substrate to form a halide perovskite crystal. Liu et al. first synthesized halide perovskites and made them into a solar cell with 15% efficiency by vapor deposition [71]. Matthew used a two-step method to synthesize halide perovskite, wherein a layer of metal halides (PbCl_2_ and PbI_2_) was deposited , followed by adding ammonium halide salts (such as methyl ammonium iodide, formazan bromide) in a dedicated area [72]. It was converted to a gas phase and then deposited on a substrate downstream of another region of the tube furnace. The halide perovskite phase synthesized by this method was greatly improved compared with the conventional stability. Tavakoil et al. synthesized halide perovskite by a one-step deposition on a c-TiO_2_-coated FTO glass substrate [73]. Tong et al. prepared a two-phase all-inorganic halide perovskite composite CsPbBr_3_-CsPb_2_Br_5_ film capable of functioning as a photodetector by vapor deposition using a controlled excess of PbBr_2_ [74]. A method of gas phase deposition preparation by Lin substituted a part of 3D-MAPbI_3_ with 2D-(BA)_2_(MA)_n-1_Pb_n_I_3n+1_ halide perovskite sheet to replace MA(CH_3_NH_3_) with a molecule of BA [75].

## 2. Halide Perovskite for Solar Cells

### 2.1. Performance of Photovoltaic Devices

In the 21st century, clean, low cost and sustainable energy is the most important scientific and technical challenge [78]. Photovoltaics (PV, or solar cells) are ideal energy conversion processes which can meet these requirements. Back in 1954 in Bell Lab, the first PV devices based on crystalline silicon (c-Si) were invented. Currently, most of the commercially available solar cells (PVs) are inorganic silicon semiconductors, either single crystal or polycrystalline silicon.

**Figure 5:**
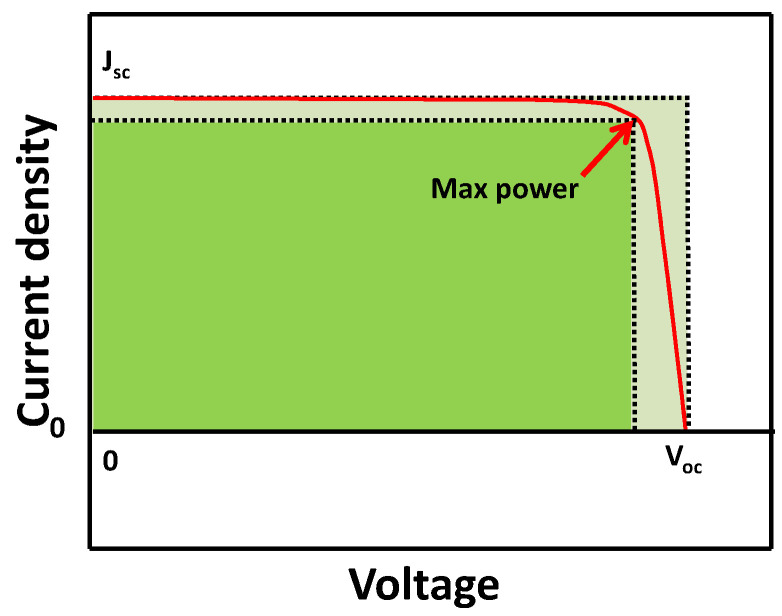
Demonstration of I-V curve, max power, and FF (fill factor) for a solar cell.

Short circuit current density (*J_sc_*, Figure 5): When the solar cell is short circuited under illumination, *J_sc_* is the photo-current per unit area (mA/cm^2^). Theoretically, *J_sc_* can be calculated from the incident photon-to-current efficiency (IPCE) spectrum. The light intensity, light absorption, and injection efficiency can affect *J_sc_*.

Open circuit voltage (*V_oc_*, Figure 5): Under the illumination of light with the circuit open, the potential between two electrodes in the solar cells/photovoltaics devices is defined as the *V_oc_*. In theory, the *V_oc_* can be as high as the difference between the semiconductor’s Fermi level and the potential of the hole (vacancy) conductor. It is measured when the current through the solar cells is zero volt (an open circuit).

Fill factor (FF, Figure 5): The FF is defined as the ratio of the maximum power output per unit area (in percentage %) to the product of *V_oc_* and *J_sc_*, which measures the ideality of the solar cells. High series resistance (or internal resistance) results in lower fill factor and correspondingly decreased overall efficiency.

Incident photon to current efficiency (IPCE): The ratio of *N_e_*/*N_p_* is defined as the monochromatic incident photon-to-electron conversion efficiency (IPCE), in which *N_e_* is the number of produced electrons in the external circuit. And *N_p_* is the number of incident photons. *λ* is the wavelength of the incident light in Equation (2), while the intensity for the incident light is *P_in_*. The units for *J_sc_*, λ, and *Φ_in_* are mA/cm^2^, nm, and W/m^2^, respectively.

*IPCE* = 1240·*J_sc_*/(*λ*·*P_in_*) (2)

Equation (3) shows the relation between IPCE, LHE (light harvesting efficiency), *η_ci_* (charge injection efficiency), and *η_c_*_c_ (charge collection efficiency on back contact):

*IPCE* = *LHE*·*η_ci_*·*η_cc_* (3)

Solar energy to electricity conversion efficiency (*η*): As the most important parameter, the overall solar energy to electricity conversion efficiency is shown in Equation (4), which is the ratio of the maximum output of the cell divided by the power of the incident light. Equation (4) shows the calculation of the *η* from *J_sc_*, *V_oc_*, *FF*, and the intensity of the incident light (*P_in_*).

*η* = *P_opt_*/*P_in_* = (*FF* ×* I_sc_* × *V_oc_*)/*P_in_* (4)

The properties of materials change significantly from bulk into nanoscale, with significant improvement in surface area, charge transport, etc. due to the quantum effect.

### 2.2. Rising of Halide Perovskite Solar Cells

#### 2.2.1. Dye Sensitized Solar Cell (DSSC) to Halide Perovskite Solar Cell

As the first generation of solar cells, silicon solar cells exhibit over 20% power conversion efficiencies (PCEs). However, the cost for production in large scale is still high due to the requirements for the processing conditions, which increase the cost. Thus, the needs for developing low cost and new types of solar cells become more and more important, such as solar cells based on thin film organic, inorganic or hybrid materials [40]. CdTe and CuInGaSe (CIGS) are considered as second generation thin film solar cells with PCE over 19.6% per cm^2^ [79]. However, both CdTe and CIGS solar cells have difficulties in large-scale production (requirement for ultra-high vacuum) and the use of expensive elements [80].

**Figure 6:**
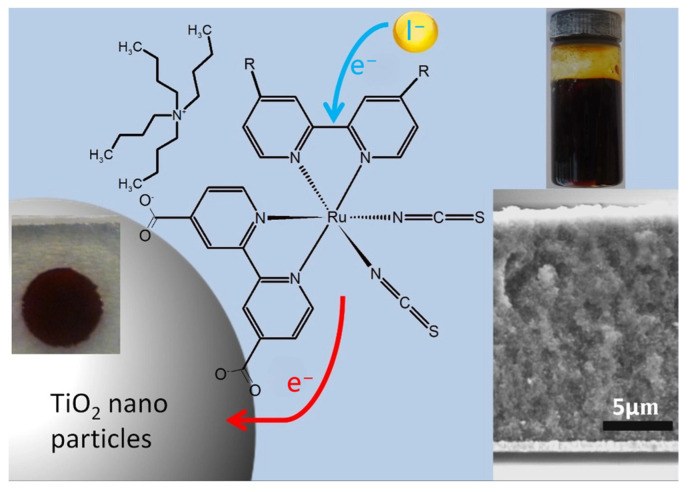
A description of DSSC with optical image and SEM image [83].

Dye-sensitized solar cells (DSSCs): Usually, mesoscopic solar cells have a low cost and are easy to fabricate, which are good candidates as low cost next generation PV devices. DSSC is a typical mesoscopic solar cell. In a DSSC, dyes as light absorber were anchored on a nanostructured TiO_2_ electron conductor, I_3_^−^ ions in electrolytes were used as redox shuttle for dye regeneration, and a counter electrode such as Pt was used to collect electrons. In the past 25 years, efforts have been made, such as the synthesis of dyes, improvement for the electron conductor, redox shuttles, fundamental understanding of the working principles, and many other aspects for DSSCs. DSSCs with over 13.0% PCE can be easily achieved at lab scale and achieved 10% in modules [81,82]. The description, SEM image, and optical image of DSSC are shown in Figure 6 [83].

Theoretically, voltage of the solar cell (*V_oc_*) is determined by the difference between the Fermi level of TiO_2_ (where dyes anchored) and the Nernst potential of the redox shuttles in the electrolytes [84,85]. However, DSSCs are still far away from large scale application, which arises from the evaporation and corrosion of liquid electrolytes. Although DSSC based on solid hole transport material (HTM) has solved the problem of liquid electrolytes, the efficiency is still low compared with liquid electrolytes and with silicon-based solar cells.

**Figure 7:**
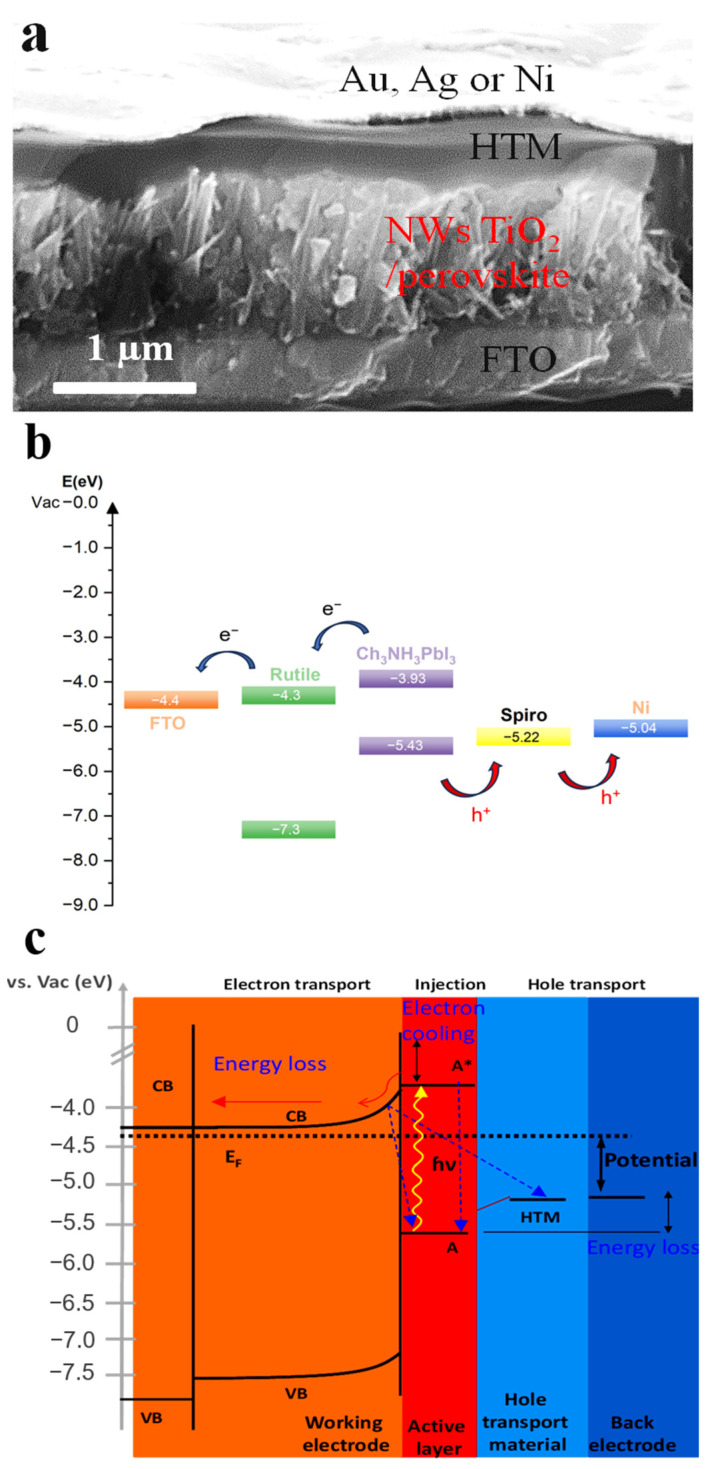
(**a**) SEM image of the cross section for a halide perovskite solar cell based on TiO_2_ nanowire [89]. (**b**) Energy diagram of halide perovskite solar cell [90]. (**c**) Mechanism and kinetic diagram (* indicates hot electron).

Optical-electronic process and charge dynamic in halide perovskite solar cells: Halide perovskite solar cells are based on DSSCs. Usually, halide perovskite solar cells have a sandwich structure like DSSC as shown in Figure 7a. A layer of TiO_2_ on FTO glass is used as the photo-electrode. CH_3_NH_3_PbI_3_ is spin-coated or dip-coated as the photo-active layer (light absorber). Then, the HTM layer such as spiro-OMeTAD with additives is spin-coated. Finally, gold, silver or other inert metals are thermo-coated (such as CVD) as the back electrode.

Figure 7b is a typical energy diagram for halide perovskite solar cells, which is similar to DSSCs: excitation, ejection, regeneration, recombination, and migration as in DSSCs.

Figure 7c shows the kinetic diagram for halide perovskite solar cells MPbX_3_ (M = CH_3_NH_3_^+^_,_ Cs^+^_,_ FA^+^ or a mixture; X = I^−^, Br^−^, Cl^−^, SCN^−^ or a mixture) [39,86,87,88,89,90,91,92,93,94]. Light with photons of energy greater than the band gap are absorbed by active layer and the electronic state changes from the ground state (A) to the excited state (A*, hot electrons). The lifetime for these hot electrons is on the order of nanoseconds. The hot electrons can be transferred in femtoseconds to the conduction band of the working electrode, usually comprising metal oxides such as TiO_2_, ZnO, and other materials with the matching band. It takes up to seconds for the electrons to be transported in the metal oxides. On the other hand, the hot electrons can recombine with the hole, which in turn cause the low efficiency.

In 2009, Kojima reported the first application of hybrid organic–inorganic solar cells based on halide perovskite CH_3_NH_3_PbI_3_ as the photon active layer with an efficiency of 3.8% [6]. In 2012, after three years, the efficiency reached 10.9% [95]. Two Nature publications were reported separately by Dr. Gräetzel and Dr. Snaith with over 15% efficiency in 2013 [71,91]. In late 2014, the efficiency for halide perovskite solar cells reached about 20% [96]. Since then, halide perovskite solar cells attracted worldwide attention in the photovoltaic community.

#### 2.2.2. Progress in Halide Perovskite Solar Cell

**Figure 8:**
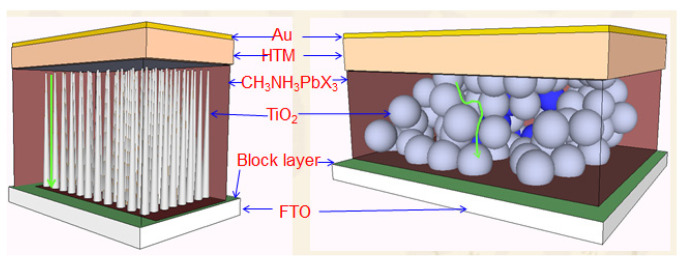
Perovskite solar cell with 3D-TiO_2_ as photo-anode (arrows indicate the path for electron) [89].

**Figure 9:**
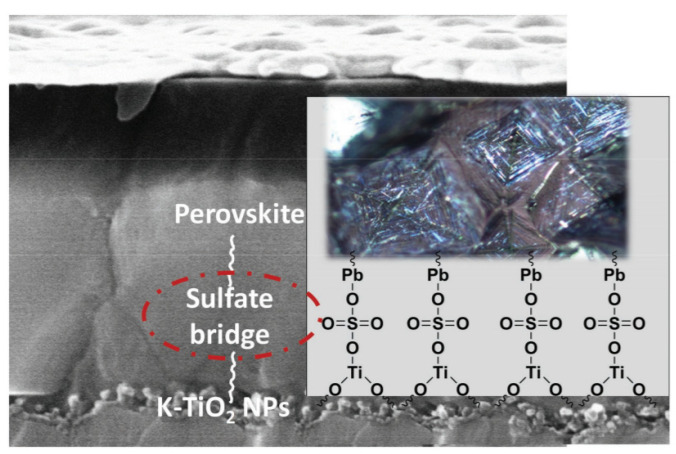
Schematic illustration of doped TiO_2_ mesoporous layer and the surface states bonding with ETL and halide perovskite [103].

3-D structure perovskite solar cell: Usually, a layer of mesoporous TiO_2_ particle is used as the photo-anode as shown Figure 8 [89,91,95,97,98]. This mesoporous 3D structure plays the role of electron transport/buffer layer, a structure supporting layer and reflection layer. ZnO can be used as a photo-anode, as well [99]. It has been reported to have an efficiency of over 11% [100]. Al_2_O_3_ can also be used as the photo-anode with an efficiency of 8.3% [101]. However, TiO_2_ is still the best electron transport material for halide perovskite solar cells. Thambidurai added Ba(OH)_2_ as an additive to modify mesoporous TiO_2_ [102]. The Ba(OH)_2_ modification altered the conduction band of mesoporous TiO_2_, resulting in better coordination with the halide perovskite level, reduced carrier recombination, enhanced optical absorption, and electron transport. Singh found that the introduction of an alkali metal dopant in mesoporous TiO_2_ can effectively regulate electron conductivity and improve the charge extraction process by balancing oxygen vacancies as a non-radiative recombination center [103]. In addition, as shown in Figure 9, the sulfate bridge (SO_4_^2−^) is grafted onto the surface of the K-doped mesoporous titania to provide seamless integration of the absorber and the electron transport layer, accelerating the overall transport kinetics. Potassium doping significantly affects the nucleation of the halide perovskite layer to produce a high density film with faceted crystallites.

Yang proposed a mechanism for the preparation of halide perovskite solar cells based on TiO_2_ nanorod arrays, revealing the intrinsic relationship between the precursor concentration and the crystallite growth of the halide perovskite film prepared by the anti-solvent quenching method [104].

**Figure 10:**
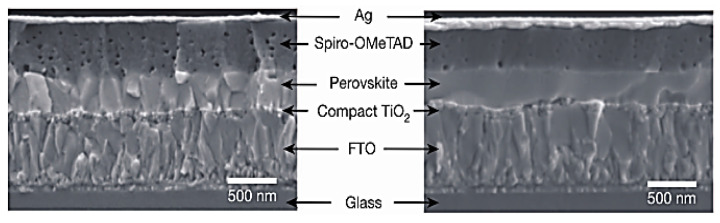
A typical planar structure of halide perovskite solar cells [71].

Planar structure halide perovskite solar cells: Planar structure halide perovskite solar cells do not have a thick electron transport layer such as mesoporous structure TiO_2_. However, a thin layer of oxides (usually TiO_2_ is less than 20 nm) is still used to block the recombination of photo-electrons [71,96]. Compact TiO_2_ is sprayed or spin-coated on TCO (transparent conductive oxide, such as FTO or ITO). A planar structure halide perovskite solar cell is shown in Figure 10 and no thick 3D oxides are used in the solar cell.

**Figure 11:**
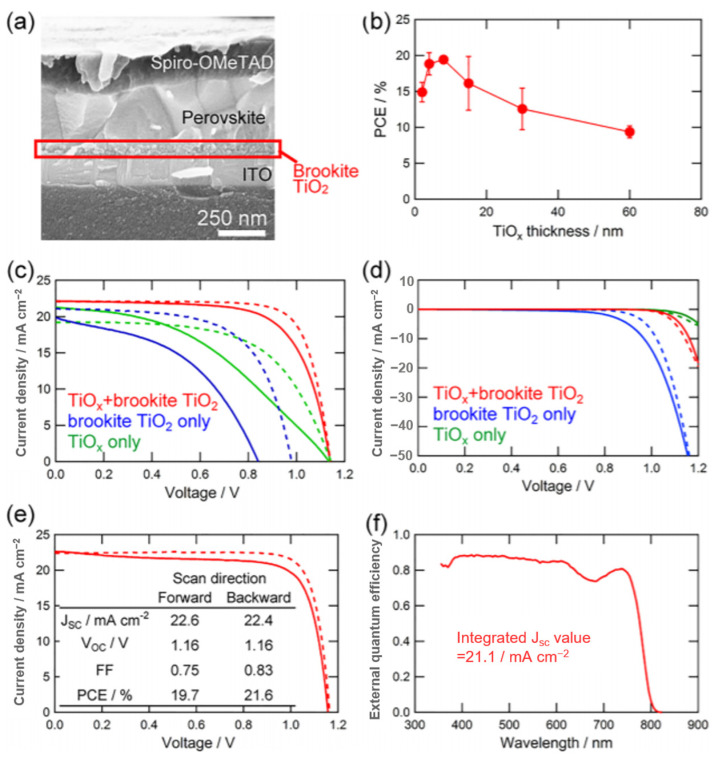
(**a**) Cross-sectional SEM image for CH_3_NH_3_PbI_3_ halide perovskite solar cells with TiO_x_ (thickness ∼8 nm)/brookite TiO_2_ as electron collector. (**b**) Dependence of PCE measured by J-V curves with 1.2 V → −0.1 V voltage scan direction on thickness of TiO_x_. J-V curves of solar cells with brookite TiO_2_ (blue), TiO_x_ (thickness ∼8 nm, green), and TiO_x_/brookite TiO_2_ (red) electron collectors measured (**c**) under 1 sun illumination and (**d**) in the dark. Forward (−0.1 V → 1.2 V) and backward (1.2 V → −0.1 V) scans are indicated as solid and dashed lines, respectively. (**e**) J-V curve and (**f**) EQE spectrum of the best solar cells with TiO_x_ (thickness ∼8 nm)/brookite TiO_2_ electron collector stored in dry air for 2 days [105].

**Figure 12:**
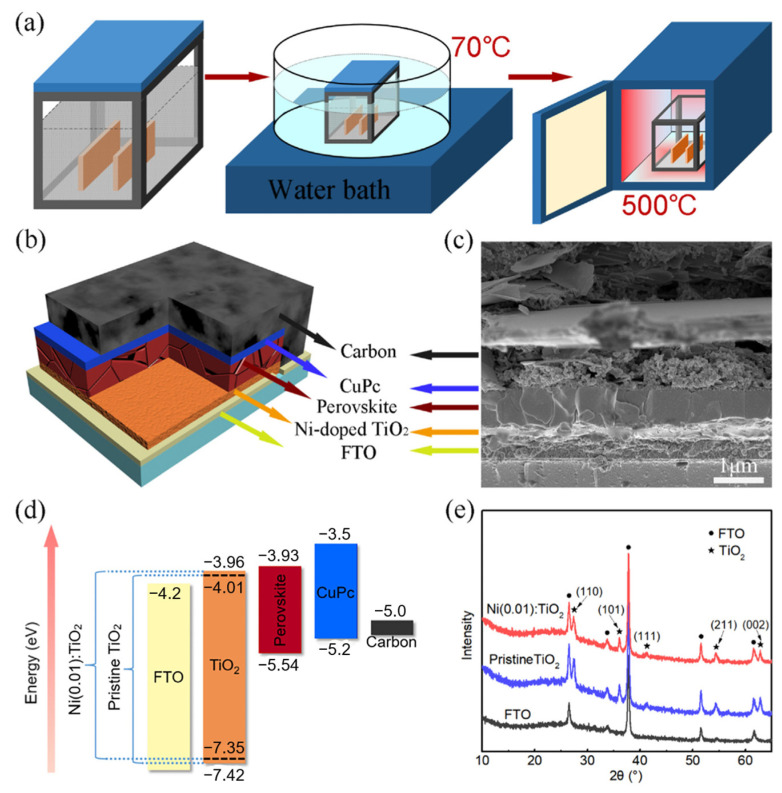
(**a**) Schematic illustration of the solution-processed method to prepare the Ni-doped TiO_2_ ETLs, including a CBD process at 70 °C and a post-annealing process at 500 °C. (**b**) Schematic illustration and (**c**) a high-resolution cross-sectional SEM image of the carbon-based planar-structured PSC. (**d**) Energy level diagram of the as-prepared PSCs. (**e**) XRD patterns of the pristine TiO_2_ and Ni(0.01):TiO_2_ deposited on FTO substrates [106].

In Figure 11, Kogo prepared a halide perovskite solar cell using an ultra-thin amorphous TiO_x_ as a hole blocking layer in combination with brookite-TiO_2_ prepared below 150 °C [105]. Consisting of a TiO_x_/brookite-TiO_2_ double layer electron collector, the halide perovskite solar cell has a high efficiency of 21.6% and a high open circuit voltage and fill factor of 1.18 V and 0.83, respectively. Liu et al. used high crystallinity Ni-doped rutile TiO_2_ as the carbon-based planar heterojunction PSC of the electron transport layer (ETL), and simultaneously introduces copper phthalocyanine (CuPc) as the hole transport layer (HTL) (Figure 12) [106]. The doping of Ni can shift the Fermi level of ETL upward and correspondingly increased the charge mobility in TiO_2_, thereby enhancing charge transport and extraction. The excellent properties of Ni-doped TiO_2_ in promoting charge transfer and suppressing carrier recombination were disclosed. In the study of pure rutile TiO_2_, Wang used rutile and anatase TiO_2_ electron transport layer (ETL) to study crystalline phase-dependent charge collection to fabricate solar cells. The rutile TiO_2_ was found to enhance electron transport to the FTO due to the better contact between the rutile TiO_2_ and the halide perovskite particles and a smaller trap density [107]. It exhibited better electrical conductivity and improved interfacial contact with the perovskite layer. The highest efficiency achieved using the rutile TiO_2_ electron transport layer was 20.9%.

**Figure 13:**
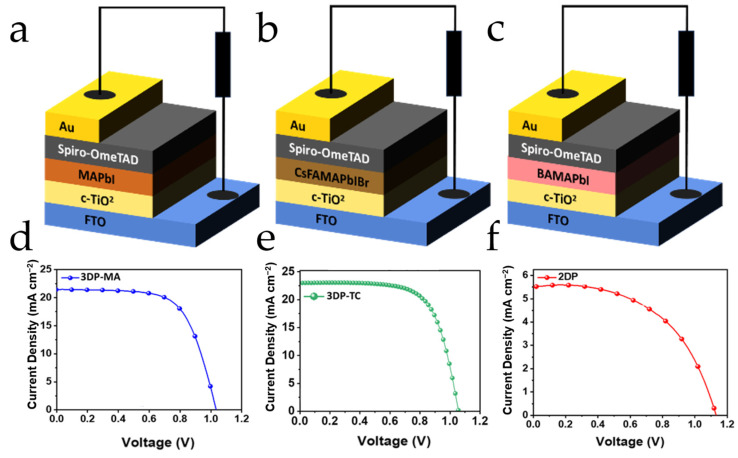
Visual representation of n-i-p architecture of (**a**) 3DP-MA, (**b**) 3DP-TC, and (**c**) 2DP. Visual representation of the respective n-i-p architectures used along with data for the champion pixels. J-V characteristic for the: (**d**) 3DP-MA absorber yielding a PCE of ~15.69%, (**e**) 3DP-TC absorber yielding a PCE of ~16.49%, and (**f**) 2DP absorber yielding a PCE of ~3.33% [108].

Kaul analyzed the potential uses of three types of halide perovskite materials in sensor and photovoltaic applications (Figure 13) [108]. These include two-dimensional halide perovskites (BA_2_MA_3_Pb_4_I_13_, 2DP), traditional three-dimensional halide perovskites (MAPbI_3_, 3DP-MA), and the more recently studied triple cation, mixed halide three-dimensional halide perovskites (Cs_0_._05_FA_0_._79_MA_0_._16_PbI_2_._45_, 3DP-TC). Reducing the dimensionality of 3DP to create two-dimensional halide perovskites (2DPs) represents a significant development [109,110]. Within this arrangement, organic spacers are sandwiched between inorganic sheets, where the general formula for 2DPs is commonly represented by *(A*′*)_m_(A)_n−_*_1_*B_n_X*_3__*n+*1_; here A′ denotes a bulky organic cation, such as aliphatic or aromatic alkylammonium, serving as a spacer between the inorganic sheets, and n represents the number of inorganic layers in the structure [111]. The study explored their photovoltaic emission properties and integrated them into an n-i-p solar cell architecture, revealing significant differences in performance metrics such as *V_oc_*, *J_sc_*, and *FF* among the three types.

**Figure 14:**
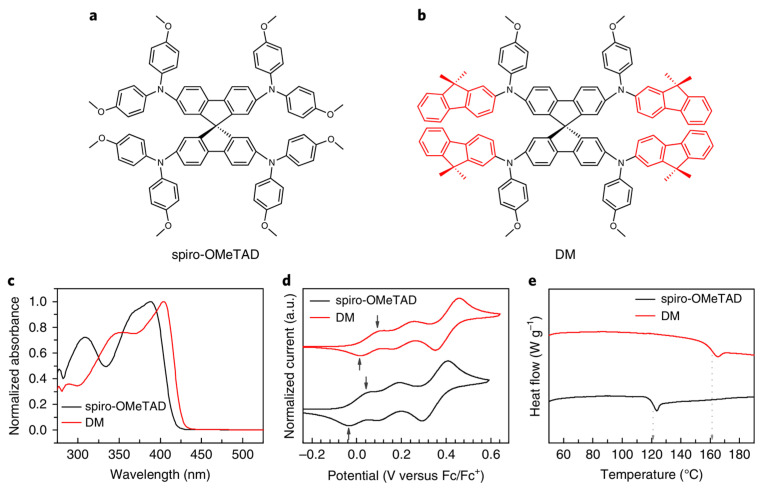
Optical, electrochemical, and thermal characteristic of HTMs. (**a**,**b**) Chemical structures of spiro-OMeTAD (**a**) and DM (**b**). (**c**) Ultraviolet-visible absorption spectra of spiro-OMeTAD and DM in the solid state. (**d**) Cyclic voltammograms (CVs) of spiro-OMeTAD and DM. The downward arrows indicate the first peak anodic potentials, and the upward arrows indicate the first peak cathodic potentials. (**e**) DSC curves of spiro-OMeTAD and DM. The vertical dashed lines indicate the glass transition temperature [117].

Hole transport materials (HTM): In a typical HTM layer of halide perovskite solar cell, spiro-OMeTAD is doped by lithium bis-trifluoromethane sulfonamide, 4-tert-butylpyridine, and other additives. Modifications of spiro-OMeTAD were carried out by a large amount of research groups. The methoxy substituents of spiro-OMeTAD were reported to enhance FF and PCE [112]. The dicationic salt for spiro-OMeTAD was discovered to improve hole conductivity [113]. Many other organic HTM materials, such as carbazole-based materials [114], thiophene-based materials [101], quinolizino acridine-based materials [115], and pyrene arylamine type materials [116] were also reported as alternatives of spiro-OMeTAD with efficiencies of 9.8%, 13.8%, 12.8%, and 12.4%, respectively. It is worth noting that Jeon synthesized a ruthenium-capped hole transport material with a fine-tuning level and a high glass transition temperature (Figure 14) [117]. Photovoltaic devices (under reverse scanning) based on this material achieved an efficiency of 23.2%. Moreover, the resulting device showed better thermal stability than devices with spiro-OMeTAD as HTM, maintaining its initial performance after thermal annealing at 60 °C, which approximately 95% of the time exceeds 500 hrs.

A few years later, inorganic materials-based HTM for halide perovskite solar cells were reported, such as CuI [118] and CuSCN [119] with efficiencies of 6.0% and 12.4%, respectively. It is worth noting that when Arora used copper thiocyanate (CuSCN) as the hole extraction layer, the stability efficiency of PSC exceeds 20% [120]. The use of a rapid solvent removal method enables compact formation and facilitates carrier extraction and collection. PSC exhibits high thermal stability under long-term heating, but their operational stability is still poor. It is believed that this instability stemmed from the potential-induced degradation of CuSCN/Au contact. After a conductive reduced graphene oxide spacer was added between CuSCN and gold, this keeps the PSC at the maximum power point and maintains >95% of its initial efficiency after aging for 1000 h at full solar intensity at 60 °C.

Although Ma Tingli (9%) [121], Meng Qingbo (10.5%) [122], and Han Hongwei et al. (12.8%) [123] reported the HTM free halide perovskite solar cells; however, the highest efficiency record for halide perovskite solar cells was still achieved by spiro-OMeTAD-based HTM.

Lead free halide perovskite solar cells: Lead is toxic and causes problems in both environment and health. Thus, the requirement for lead free halide perovskite solar cells have been proposed. Tin, which is in the same group as lead, has enhanced absorption in red light and NIR (near infrared), and it has been used to replace the lead in the halide perovskite solar cells. However, the efficiency is low compared with lead-based solar cells [124,125]. Reports show that the reasons for the low efficiency are due to the decreased absorption in visible light and shift of valence band [126].

Engineering for halide perovskite crystals: The morphology and quality of the halide perovskite material crystal is the key for high efficiency halide perovskite solar cells. There are many factors that can affect the crystallinity quality of halide perovskite materials, such as spin-coating temperature and speed [127], concentration of the precursor [128], annealing temperature and time [129,130], moisture [14], and solvents [131], which in turn affect the efficiency of halide perovskite solar cells. Different types of halide perovskite crystals can be synthesized, such as cubic particles, hexagonal particles, and nanorods [132]. In Table 1, the studies that optimize the crystallinity quality of halide perovskite materials have been summarized.

With the yearly progress of an increase in efficiency (Table 2), many efforts have been made to improve the current density (*J_sc_*), open circuit voltage (*V_oc_*), fill factor (*FF*), and efficiency by the crystals engineering for halide perovskite materials and replacement of the costly parts in the solar cells, such as the noble gold or silver back electrode and hole transport materials (HTMs).

Stability of halide perovskite solar cells: Another big issue is the stability of halide perovskite solar cells. Halide perovskite materials can be destroyed by the moisture and the HTM based on spiro-OMeTAD can be oxidized easily in the air. Table 3 shows the recent progress to improve the stability of halide perovskite solar cells.

Replacement and Yttrium doped TiO_2_: It has been reported that the work function of TiO_2_ ETL can be reduced by Y doping, and therefore enhanced the electron extraction and transport channel. Y-doped TiO_2_ devices exhibited faster photo-current decay than that of reference devices in the device [96].

Ni doped Fe_2_O_3_: The addition of nickel (Ni) dopants can enhance the electron conduction and induce a downward shift of the CB minimum for α-Fe_2_O_3_. In turn, it facilitated electrons injection and transfer from the CB of halide perovskite materials. Thus, a substantial reduction in the charge accumulation at the halide perovskite material/ETL interface makes the solar cells much less sensitive to scanning rate and scan direction during the efficiency measurement, to be specifically the lower hysteresis. Meanwhile, solar cells with good stability when exposed to air and high levels of ultraviolet (UV) light can be achieved [199].

### 2.3. Challenges in Halide Perovskite Solar Cells

#### 2.3.1. Stability of Halide Perovskite and Spiro-OMeTAD

**Figure 15:**
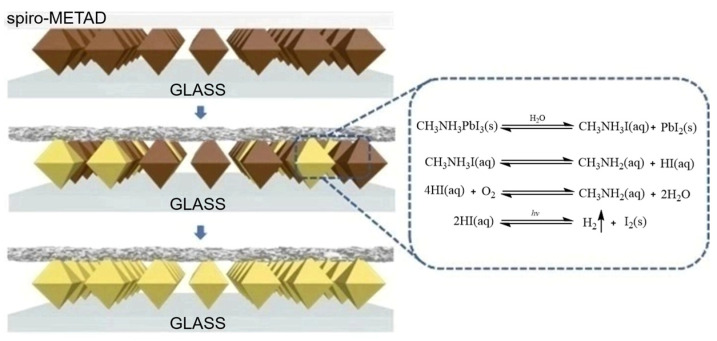
Decomposition of active layer in halide perovskite solar cell [201].

Amazing progresses have been made for halide perovskite solar cells; however, the dark sides cannot be ignored such as the stability [200]. The main barrier for the commercialization of halide perovskite solar cells is the decomposition of halide perovskite materials when exposed to air [201,202,203]. There are efforts to improve the stability of the halide perovskite materials, such as using FAPbI_3_ to replace MAPbI_3_ [92,204] and changing the morphology of halide perovskite materials [205]. The decomposition of halide perovskite materials is shown in Figure 15 [201].

#### 2.3.2. Light Harvesting vs. Charge Transport

To obtain high photo-current (*J_sc_*), a thick layer of photo-active materials is required to harvest as much light as possible. On the other hand, it requires fast charge transport and a short diffusion distance to avoid the recombination of photo-electrons and holes. Both the *V_oc_* and *FF* drop when the thickness is over 600 nm for halide perovskite solar cells due to the increase in dark current and electron transport resistance [40]. The optimized thickness for light-active layer in perovskite solar cells is between 400 and 600 nm to avoid the drop in *V_oc_* and *FF* [91]. In the halide perovskite solar cells based on mesoporous TiO_2_ films, filling the highly convoluted porous channels of halide perovskite CH_3_NH_3_PbX_3_ solar cells is very difficult [206]. Meanwhile, the light-harvesting layer is quite thin for high efficiency (mainly photo-current) due to the electron diffusion length in halide perovskite CH_3_NH_3_PbI_3_, which is about 100 nm [41]. It has been well studied that if the mesoporous TiO_2_ is over 600 nm, there will be a significant drop in *V_oc_* and *FF* [40]. Thus, the optimized thickness for mesoporous TiO_2_ layer is between 400 and 600 nm [91,115,124,150].

#### 2.3.3. Even Lower Cost for HTM and Counter Electrode

The use of costly complex organics in the hole transport layer (HTM) can be substituted with more affordable inorganic materials to reduce expenses [118,119]. Furthermore, the organic compounds in HTM decompose in UV light and high temperature.

In order to obtain the attainable *V_oc_*, expensive metals such as gold [91,101,150] and silver [71,207] with low chemical potential and high work function are commonly used as the counter electrode. Thermal evaporation (such as PVD) of gold is a costly and wasteful process since only a small portion of gold is deposited onto the solar cells. Thus, replacing gold with cheaper elements for halide perovskite solar cells while keeping the high *V_oc_* is critical to reduce the cost for halide perovskite solar cells.

## 3. Halide Perovskite for Light Emitting

### 3.1. Basics of Light Emitting

An LED is a device that radiates visible light when electrons and holes recombine, and is a diode composed of a p-type and an n-type semiconductor. The principle of light emission is that the anode is injected into the cavity and the cathode is injected with electrons. The holes and electrons are respectively passed through the transport layer and finally the exciton light is formed in the light emitting layer [6]. In the LED, the PN junction is applied with an electric field luminescence, and as electrons cross from the N region and recombine with the holes present in the P region, the charge carriers recombine in the forward biased PN junction. The free electrons are in the conduction band of the energy level, and the holes are in the valence band energy level. Therefore, the energy level of the holes is lower than the energy level of the electrons. Some of the energy must be dissipated to recombine electrons and holes. This energy is emitted in the form of heat and light. It releases excitons with an energy of hν. Additionally, the energy corresponds to the band gap energy E_g_ of the semiconductor material, and the relationship of the emission wavelength λ(nm) is:

(5) $$\lambda =1239.6/E_{g}$$

In the study of LED devices, external quantum efficiency (*EQE*) is a very critical parameter. When the photon is incident on the surface of the halide perovskite material, part of the photons will excite the halide perovskite material. At the same time, electron–hole pairs are generated, thereby forming a current which results in arranging the ratio of the collected electrons to the number of all incident photons. Its value can be calculated by the formula (Equation (6)):

(6) $$\mathrm{EQE}\;={\;\mathrm{IQE}*\eta }_{0}$$

where IQE is the internal quantum efficiency and η_0_ is the ratio of photons emitted to free space.

Other parameters for LED devices, such as power efficiency (*PE*), current efficiency (*CE*), turn-on voltage (*V_on_*), maximum brightness (*L_max_*), and stability are also critical parameters for LEDs. Among them, power efficiency and current efficiency can be calculated by (Equations (7) and (8)):

(7) $$\mathrm{EQE}\;={\;\mathrm{IQE}*\eta }_{0}$$

**Figure 16:**
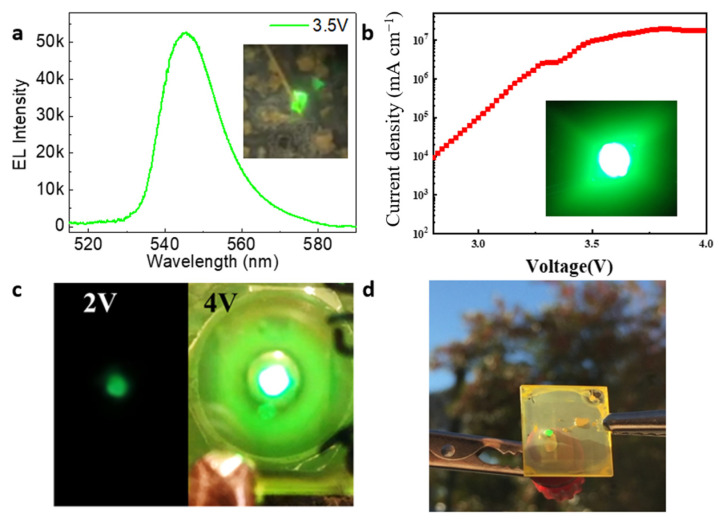
(**a**) Electroluminescence spectrum and a microscopic optical image from a light emitting device with the halide perovskite [208]. (**b**) Current density of green light emitting device [209]. Both (**c**) and (**d**) light emitting devices operating in different conditions [210].

(8) CE=L/J

where P is the power of photon emission into free space, L is the brightness of the LED, and J is the current density. Figure 16 showed an example of light emitting devices.

### 3.2. Multi-Layer Halide Perovskite Light Emitting Device

Multi-layer halide perovskite LED devices have evolved from halide perovskite solar cells, generally consisting of a perovskite layer, multiple transport layers, a metal electrode, and an ITO composition.

In multi-layer halide perovskite LEDs, the interface between the halide perovskite emissive layer and the transport layer is an important factor affecting device performance. The progress and efficiency of multi-layer halide perovskite devices and their structure were summarized in Table 4.

**Figure 17:**
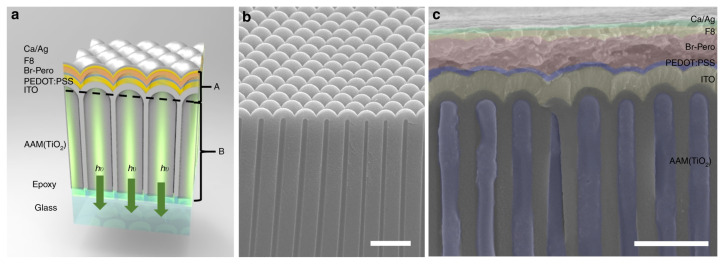
Device on nanophotonic substrate. (**a**) Schematic device. The materials from top to bottom are: Ca/Ag electrode, F8, CH_3_NH_3_PbBr_3_(Br-Pero), PEDOT: PSS, ITO, and anodic alumina membrane (AAM). AAM channels are filled with TiO_2_. (**b**) SEM image of the barrier side of the free-standing AAM film with nanodome structures. (**c**) Cross-sectional SEM image of a P500 AAM device. Scale bars in (**b**,**c**) are 1 μm [212].

Lee reported various conjugated polyelectrolytes (CPEs) as hole injection layers of MAPbBr_3_ LED, and found that PCPDT-K can effectively transfer holes, prevent electron transfer from halide perovskite to the underlying ITO layer, and reduce MAPbBr_3_/PCPDT-Fluorescence quenching at the K interface [211]. In Figure 17, Zhang et al. proposed a new device structure for the MAPbBr_3_ LED device [212]. The authors improved nanophotonic substrates and fabricated MAPbBr_3_ LEDs on them, since nanophotonic substrates were a combination of ND optocouplers and NW photonic crystal optical antennas. With these two optical components, the authors significantly improved the light extraction rate, achieving an external quantum efficiency of 17.5% for the device.

**Figure 18:**
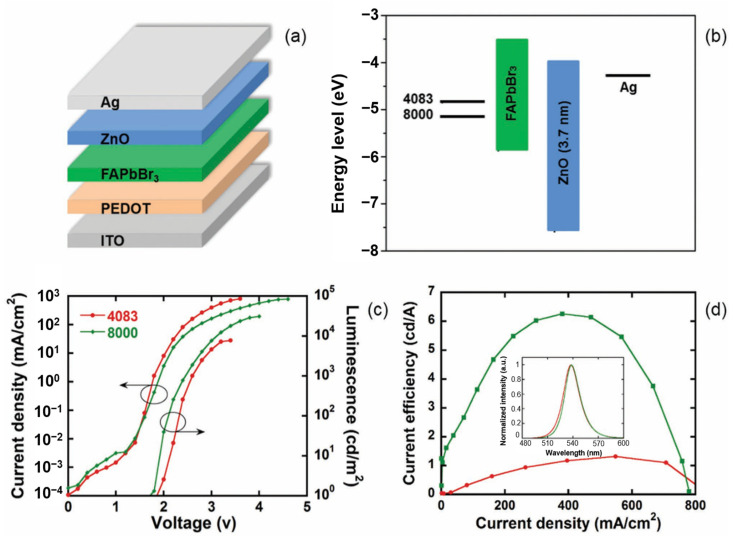
Schematic illustration of (**a**) the device structure and (**b**) the energy band diagram. (**c**) J-V-L characteristics (arrows indicate the *Y*-axis) and (**d**) LE-J characteristics of FAPbBr_3_-based PeLEDs with different HILs. Inset: EL spectra of PeLEDs recorded at 10 mA/cm^2^ [213].

Wang (Figure 18) found through experiments that the FAPbBr_3_ device exhibited hole-dominated characteristics [213]. To achieve charge carrier balance, on the anode side, PEDOT:PSS 8000 was used as the hole injection layer. Meanwhile, on the cathode side, solution-treated ZnO nanoparticles (NPs) were used as the electron injection layer in the conventional LED to improve the electron current. The prepared device achieved an EQE of 4.66% and a luminous intensity of 10,900 cd/A.

**Figure 19:**
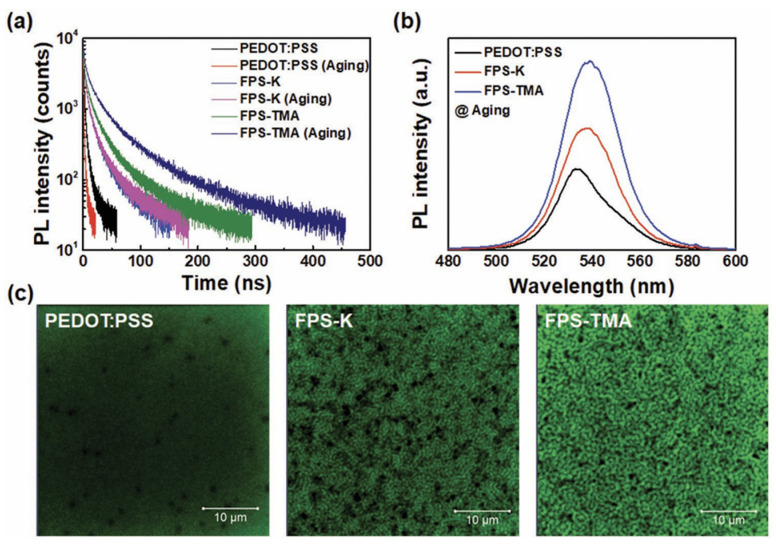
Optical properties of 120 nm-thick perovskite films deposited on PEDOT:PSS, FPS-K, and FPS-TMA before and after the aging process. (**a**) Time-resolved PL decay profiles of the perovskite films before and after aging. (**b**) Steady-state PL spectra of the perovskite films after aging. (**c**) Confocal PL images of the perovskite films after aging [215].

Yang found that the organic small molecule TOPO may be a good passivating agent [214]. It spin-coated TOPO on the surface of PEA_2_(FAPbBr_3_)_2_PbBr_4_ and found that PLQY increased significantly from 57.3% to 73.8%, and the lifetime increased from 0.17 μs to 0.36 μs. In Figure 19, Lee et al. further found that two CPEs with different counter ions could be used as multifunctional passivation and hole transport layers [215]. These layers can block opposite charges simultaneously and result in fewer interface defects in the PEA_2_ (FAPbBr_3_)_2_PbBr_4_ layer.

**Figure 20:**
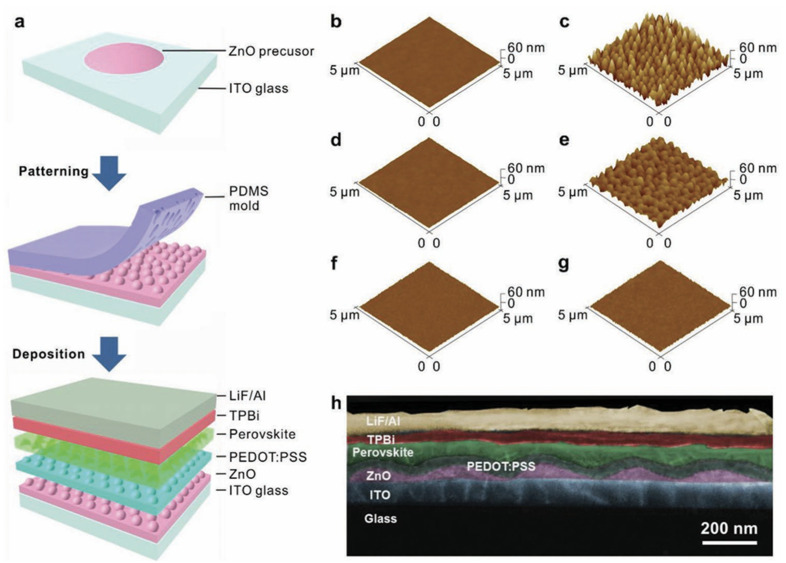
Device fabrication and film morphologies. (**a**) Schematic illustration of the fabrication process of a CsPbBr_3_ PeLED with the imprinted nanostructures. Diagrams are not to scale. (**b**–**g**) AFM images of flat (**b**) and patterned (**c**) ZnO layers on ITO-glass substrates, the PEDOT:PSS layers on flat (**d**) and patterned (**e**) ZnO substrates, and the CsPbBr_3_ perovskite films on flat (**f**) and patterned (**g**) PEDOT:PSS/ZnO substrates. (**h**) Cross-sectional SEM image of the patterned CsPbBr_3_ PeLED [36].

Liu et al. used an amphiphilic conjugated molecule, betaine, as an interfacial buffer layer [216]. It was found to be able to control the grain size of the perovskite, thereby increasing the crystal nucleation sites and significantly reducing current leakage. As a result, the device achieved a high EQE of 11%. Shen reported a bio-inspired new structure using moth-eye nanostructures at the front electrode/perovskite interface to enhance the out-coupling efficiency of waveguide light in LEDs (Figure 20) [36]. The maximum external quantum efficiency and current efficiency of the modified CsPbBr_3_ were increased to 20.3% and 61.9 cd/A. The hemispherical lens was then used to further reduce the light loss in the substrate mode, achieving an efficiency of 28.2% and 88.7 cd/A, which is currently the highest efficiency.

NiO has been extensively studied in LEDs as a hole transport material. However, using NiO nanotubes in LEDs is not common. NiO_x_ has several advantages: high carrier mobility, good stability, and processability. In contrast to its organic counterparts, NiO_x_ does not have high hygroscopicity and acidity, thus it does not damage conductive glasses like FTO and ITO. Moreover, NiO_x_ matches the energy levels of halide perovskites, making it an ideal choice for halide perovskite-based LEDs. Lin et al. proposed a novel LED approach by encapsulating MAPbBr_3_ in nickel oxide nanotubes (NiO_x_) [217]. This unique structure led to efficient electroluminescence, with significantly improved current efficiency (5.99 Cd/A) and external quantum efficiency (3.9%).

**Figure 21:**
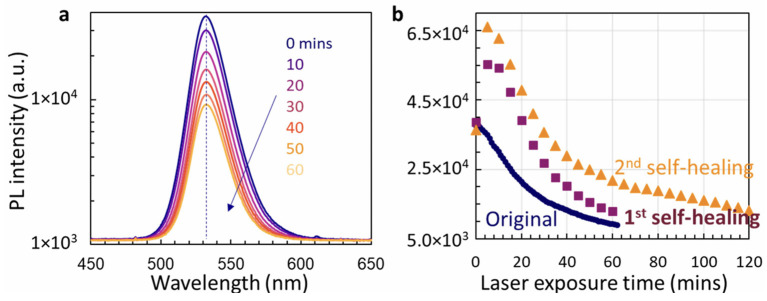
(**a**) Selective PL spectra of original MAPbBr_3_@NiO measured at 0, 10, 20, 30, 40, 50, and 60 min after the sample was exposed to the laser. (**b**) PL intensity as a function of laser exposure times for the original sample (blue symbols), the 1st PL measurement of self-healed samples after UV 375 nm laser is off overnight (purple squares), and the 2nd PL measurement of self-healed samples is second overnight (orange triangles) [218].

Lin (Figure 21) also utilized photoluminescence (PL) to study the laser radiation hardening and self-healing properties induced in aged MAPbBr_3_ halide perovskites encapsulated in NiO nanotubes (MAPbBr_3_@NiO) [218]. The study found that even after two years of exposure to atmospheric conditions, the aged samples remained highly stable. They demonstrated no change in PL wavelength during UV laser irradiation and self-healing. Additionally, UV light exposure at 375 nm enhanced the PL of the self-healed MAPbBr_3_@NiO. They also used FLIM analysis to understand the mechanisms behind photo-degradation, self-healing, and PL enhancement. They suggested that photo-degradation could be explained by the formation of numerous low-lifetime trapping states, while the enhanced PL can be attributed to the prolonged peak lifetime observed in the lifetime histogram of self-healed MAPbBr_3_@NiO.

Tang modified the FAPbI_3_/ZnMgO interface by introducing a Lewis base diamine molecule (EDBE) on top of the ZnMgO electron transport layer (ETL) [9]. With two amino groups in EDBE, one amino group can interact with ZnMgO to adjust the growth of the perovskite film, thereby improving electron injection and suppressing current leakage. At the same time, another amino group can passivate the trap state on the surface of the poly crystalline halide perovskite and eliminate trap-mediated non-radiative recombination.

**Figure 22:**
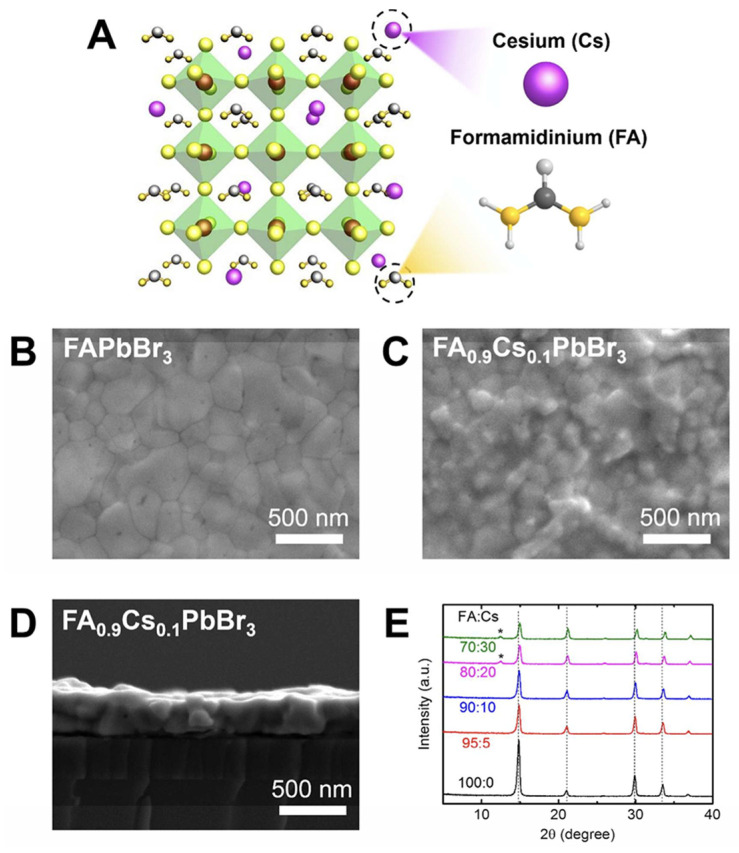
(**A**) The structure of MHPs crystal based on FA^+^ and Cs^+^. SEM images of (**B**) pure FAPbBr_3_ and (**C**) FA_0_._9_Cs_0_._1_PbBr_3_ polycrystalline films. (**D**) SEM image of a cross section of a FA_0_._9_Cs_0_._1_PbBr_3_ polycrystalline film on an SOCP layer. (**E**) XRD patterns of FA_1−x_Cs_x_PbBr_3_ polycrystalline films with various FA:Cs molar ratios (curves are offset for clarity,* show new peaks) [220].

The study of the halide perovskite emission layer itself is also a hot topic. Prakasam reported that the reduction in the thickness of the MAPbBr_3_ layer and the increase in the ratio of MABr to PbBr_2_ during synthesis can reduce the crystallite size and surface roughness [219]. The device balanced charge injection, space charge limitation, and reduction in non-radiative sites, resulting in improved device performance. Cho (Figure 22) doped Cs^+^ in FAPbBr_3_ halide perovskite, which can significantly reduce the average grain size and trap density [220]. However, as the Cs molar ratio further resulted in decreasing crystallinity and purity, trap density increased and efficiency reduced.

**Figure 23:**
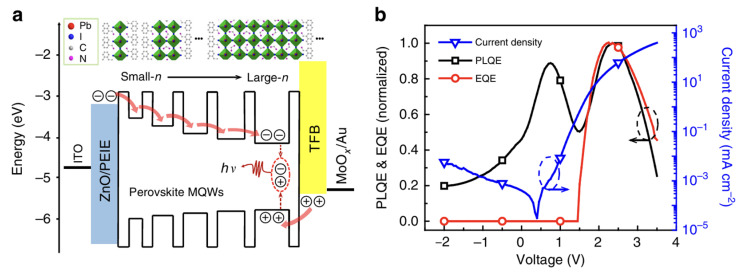
Device structure and efficiency roll-off of halide perovskite MQW LEDs. (**a**) Schematic representation of the flat-band energy level diagram and structures of the 30 nm thick halide perovskite MQW film. (**b**) Dependence of current density (blue triangles), normalized PLQE (black square), and EQE (red circle) on the driving voltage [223].

Wu et al. added small basic ions such as Na^+^ to replace the long organic molecules in the inorganic–organic perovskite to form a microcrystalline orientation [221]. The authors also found that the incorporated Na^+^ salt produced amorphous NaPbBr_3_, which was able to form a nanocrystalline halide perovskite film as a spacer in the halide perovskite, enhancing the photoluminescence lifetime. The final device achieved a high EQE of 15.9%. Cao was capable of spontaneously forming sub-micron structures by introducing amino acid additives into the perovskite precursor solution of methyl methoxide as a cation, resulting in an astonishing 20.7% EQE [222]. Studies have shown that additives can effectively passivate surface defects of halide perovskites and reduce non-radiative recombination, thereby improving efficiency. In Figure 23, Zou found that by adjusting the proportion of large and small organic cations in the precursor solution, it was easy to increase the width of the quantum well in the halide perovskite, reduce the non-radiative Auger recombination, and reduce the fluorescence quenching to improve the efficiency [223].

In Figure 23b, the PLQE and EQE were measured simultaneously on a working LED device. The excellent correlation between the PLQE and EQE at high current intensities indicates that luminescence quenching is responsible for the EQE roll-off [223].

**Table 4:** Halide perovskite LED performance.

Years	Perovskite	Type	EQE	PLQY	EL	L_max_	CE	Device Structure	Ref.
**Inorganic** **BLUE**									
**2015.10**	CsPb(Br_x_Cl_1-x_)_3_	QDs	0.07		455	742		ITO/PEDOT:PSS/PVK/CsPb(Br_1−x_Cl_x_)_3_/TPBi/LiF/Al	[47]
**2018.03**	CsPbBr_x_Cl_3−x_	3D	0.5		469			ITO/Pedot/TFB/PFI/CsPbBr_x_Cl_3−x_/TPBi/LiF/Al	[224]
**2018.05**	CsPbBr_3_	2D	0.1				25	ITO/PEDOT:PSS/Poly-TPD/CsPbBr_3_/TPBi/LiF/Al	[225]
**2019.05**	CsPb(Br/Cl)_3_	3D	1.4		463			ITO/PEDOT:PSS/PolyTPD/CBP/CsPb(Br/Cl)_3_/B3PYMPM/LiF/Al	[226]
**2021.04**	CsPbBr_3-x_Cl_x_	3D	1.18		490	1468		ITO/PEDOT:PSS/CsPbBr_3-x_Cl_x_/TPBi/LiF/Al	[227]
**2021.11**	CsPbBr_2_Cl	3D	3.71	66.8	475	51		ITO/Glass/ CsPbBr_2_Cl/TPBi/LiF/Al	[228]
**2022.10**	CsPb(Br_0_._65_Cl_0_._35_)_3_	3D	4.6		468	1680		ITO/PEDOT:PSS/CsPb(Br_0_._65_Cl_0_._35_)_3_/TPBi/LiF/Al	[229]
**2023.09**	CsPbBr_3_	3D	12		463	2100		ITO/PEDOT:PSS/PVK/ CsPbBrCl_3_/CNT2T/LiF/Al	[230]
**GREEN**									
**2016.04**	CsPbBr_3_-CsPb_2_Br_5_	QDs	2.21		527	3853	8.98	ITO/PEDOT:PSS/ CsPbBr_3_CsPb_2_Br_5_/TPBi/Al	[231]
**2016.11**	CsPbBr_3_	QDs	6.27		515	15,000		ITO/PEDOT:PSS/poly-TPD/ CsPbBr_3_/TPBi/LiF/Al	[232]
**2017.05**	CsPbBr_3_	QDs	8.73	42	512	1660		ITO/PEDOT:PSS/poly-TPD/ CsPbBr_3_/TPBi/LiF/Al	[233]
**2017.06**	CsPbBr_3_	3D	1.37		522	13,752	5.39	FTO/Buf-HILs/CsPBBr_3_/TPBi/LiF/Al	[234]
**2017.06**	CsPbBr_3_	QDs	1.194		515	12,090	3.1	ITO/PEDOT:PSS/poly-TPD/ CsPbBr_3_/TPBi/LiF/Al	[235]
**2017.07**	CsPbBr_3_	3D			527	10,700	2.9	ITO/PEDOT:PSS/CsPbBr_3_/TPBi/LiF/Al	[236]
**2017.10**	Cs_2_PbBr_5_	2D	1.1		520	7317		ITO/PEDOT:PSS/Cs_2_PbBr_5_/TPBi/LiF/Al	[237]
**2018.01**	CsPbBr_3_	QDs	3.79			6093.2	7.96	ITO/NiO/CsPbBr_3_/ZnO/Al	[238]
**2018.02**	CsPbBr_3_	QDs	4.626			10,206	8.736	In/ZnO/MgZnO/CsPbBr_3_/NiO/Au	[239]
**2018.05**	CsPbBr_3_	3D	2.99			~13,000	10.5	ITO/LiF/CsPbBr_3_/LiF/Bphen/LiF/Al	[240]
**2019.02**	CsPbBr_3_	2D	11.1		512		40.4	ITO/PEDOT:PSS/PVK/Betaine/ CsPbBr_3_/TPBi/LiF/Al	[216]
**2019.04**	CsPbBr_3_	3D	28.2				88.7	ITO/ZnO/PEDOT:PSS/CsPbBr_3_/TPBi/LiF/Al	[36]
**2021.07**	CsPbBr_3_	3D			531			n-ZnO/Al_2_O_3_/CsPbBr_3_/p-GaN	[241]
**2022.03**	CsPbBr_3_	3D	2.7			21,815		ITO/ZnO/Al_2_O_3_/PEIE/perovskite/polyTPD/MoO_3_/Au	[242]
**2023.07**	CsPbBr_3_	2D	4.87	5	512	7143		ITO/PEDOT:PSS/CsPbBr_3_/TPBi/LiF/Al	[243]
**RED**									
**2017.01**	CsPbI_3_	2D	10.4		750		0.22	ITO/PVK/BAI:MAPbBr_3_/TPBi/LiF/Al	[244]
**2018.02**	α-CsPbI_3_	3D	5		695			ITO/ZnO:PEIE/α-CsPbI_3_/Poly-TPD/WO_3_/Al	[245]
**2018.10**	α-CsPbI_3_	3D	8.65		682	210		ITO/PEDOT:PSS/PVK/α-CsPbI _3_/TPBi/LiF/Al	[246]
**2021.10**	CsPbI_3_	3D	13			1858		ITO/ZnO/PNCs/TCTA/MnO_2/_Ag	[247]
**2022-12**	CsPbI_3_	QDs	18			800		ITO/PEDOT:PSS+PFI/Poly-TPD/PEA-I/QDs/PO-T2T/LiF/Al	[248]
**Organic**									
**BLUE**									
**2016.05**	MAPb(BrCl)_3_	QDs	1.38		445	2673	4.01	ITO/PEOT:PSS/PVK/MAPb(BrCl)_3_/TPBi/LiF/Al	[249]
**2016.06**	(PEA)_2_PbBr_4_	2D	0.04		410			ITO/PEDOT:PSS/ (PEA)_2_PbBr_4_/TPBi/Ca/Al	[250]
**2018.08**	PEA_2_ A_1_._5_Pb_2_._5_ Br_8_._5_	2D		88	477	2480		ITO/PEDOT:PSS/ PEA_2_A_1_._5_Pb_2_._5_Br_8_._5_/TPBi/LiF/Al	[251]
**2021.09**	PFNBr	3D	11.2	82	485	3377		ITO/PVK/PFNBr/PO-T2T/Lig/Al	[252]
**2023.06**	GA_0_._1_Rb_0_._1_Cs_0_._8_PbBr_2_Cl	3D	1.5		469			ITO/PEDOT:PSS/GA_0_._1_Rb_0_._1_Cs_0_._8_PbBr_2_Cl/TPBi/LiF/Al	[253]
**2023.07**	PEA	2D	10.6		494			ITO/PEDOT:PSS+K_2_SO_4_ /PVP/PEA/TPBi/LiF/Al	[254]
**GREEN**									
**2014.08**	MAPbBr_3_	3D			517	154	0.3	ITO/PEDOT:PSS/MAPbBr_3_/F8/Ca/Ag	[255]
**2015.01**	MAPbBr_3_	3D	0.0065		515	21		ITO/PEDOT:PSS/TPD/MAPbBr_3_/Ag	[256]
**2015.02**	MAPbBr_3_	3D	3.5		532	~20,000		ITO/ZnO/PEI/TFB/MoOx/Au	[257]
**2015.02**	MAPbBr_3_/PIP	3D	1.2		532	200		ITO/PEDOT:PSS/MAPbBr_3_-PIP/F8/Ca/Ag	[258]
**2015.03**	MAPbBr_3_	3D	0.1		536	1000		(ITO)/PEDOT:PSS/MAPbBr_3_ /TmPyPB/LiF/Al	[259]
**2015.07**	MAPbBr_3_/PEO	3D			532	4064	0.74	ITO/PEO-MAPbBr_3_/Au	[260]
**2015.10**	MAPbBr_3_	3D			540	~10,000	42.9	SOCP/MAPbBr_3_/TPBI/LiF/Al	[261]
**2015.12**	MAPbBr_3_	QDs	1.1	92	525		4.5	ITO/PEDOT:PSS/MAPbBr_3_/TPBi/CsF/Al	[262]
**2015.12**	MAPbBr_3_/PEO	3D	1.1		545	21,014	4.91	ITO,CNT/PEO,MAPbBr_3_/AgNWs	[263]
**2016.04**	MAPbBr_3_	3D	0.43		536	~5000		ITO/ZnO/MAPbBr_3_/TFB/MoOx/Au	[264]
**2016.08**	CH_3_NH_2_-MAPbBr_3_	3D			550	65,300	15.9	ITO/NiO_x_/MAPbBr_3_/TPBi/LiF/Al	[265]
**2017.04**	MAPbBr_3_:PVK	QDs	2.28		512	7263	9.45	ITO/PEDOT:PSS/MAPbBr_3_:PVK/TPBi/Cs_2_CO_3_/Al	[266]
**2017.05**	PEA_2_(MA)_4_Pb_5_Br_16_	2D	7.4	60		8400		ITO/PEODOT:PSS /PEA_2_(MA)_4_Pb_5_Br_16_/TPBi/LiF/Al	[267]
**2017.08**	FAPbBr_3_	QDs	2.05		530	278	9.16	ITO/PEDOT:PSS/FAPbBr_3_/TPPi/LiF/Al	[268]
**2018.02**	PEA_2_(FAPbBr_3_)_n-1_PbBr_4_	2D	14.36	73.8			62.4	ITO/PEDOT:PSS/PEA_2_(FAPbBr_3_)_n-1_PbBr_4_ /TPBi/LiF/Al	[214]
**2018.03**	(OA)_2_(FA)_n-1_ Pb_n_Br_3n+1_	2D	13.4		530	34,480	57.6	ITO/PEDOT:PSS/(OA)_2_(FA)_n-1_Pb_n_Br_3n+1_ /TPBi/PO-T2T/Ca/Al	[269]
**2018.03**	MAPbBr_3_	3D	12.1			55,400	55.2	ITO / PEDOT:PSS/MAPbBr_3_/TPBi/LiF/Al	[270]
**2018.04**	MAPbBr_3_	QDs	12.9		524	22,830		ITO/PEDOT:PSS/MAPbBr_3_ /TPBi/B3PYMPM/Cs_2_CO_3_/Al	[271]
**2018.05**	FAPbBr_3_	3D	5.53			9472	20.3	ITO/LiF/FAPbBr_3_/LiF/Bphen/LiF/Al	[240]
**2018.05**	MAPbBr_3_	3D	2.36			36,854	8.67	ITO/LiF/MAPbBr_3_/LiF/Bphen/LiF/Al	[240]
**2018.05**	MAPbBr_3_	3D	5.66			18,100	25.97	ITO/CPEs/MAPbBr_3_/TPBi/LiF/Ag	[211]
**2018.08**	FAPbBr_3_	3D	4.66			10,900	21.3	ITO/PEDOT/FAPbBr_3_/ZnO/Ag	[213]
**2018.09**	PEABr	2D-3D	15.5	78				ITO/Poly-TPD/PEABr/TPBi/LiF/Al	[272]
**2018.11**	FAPbBr_3_	3D	11.3		535	79,700		ITO/Poly-TPD/FAPbBr_3_/TPBi/Al	[273]
**2018.11**	MAPbBr_3_	3D	3.9			17,600		ITO/PEDOT/Di-NPB/MAPbBr_3_ /BmPyPhB/LiF/Al	[219]
**2019.01**	MAPbBr_3_	3D	0.17			1260	0.79	PDZ/MAPbBr_3_/SPW-111/PFN/AgNW	[274]
**2019.01**	MAPbBr_3_	3D					9.2	VHB/PI/AgNWs/PEDOT:PSS/PVK /MAPbBr_3_/TPBi/CsF/Al	[275]
**2019.02**	MAPbBr_3_	3D	17.5					AAM/ITO/PEDOT:PSS/MAPbBr_3_ /F8/Ca/Ag	[212]
**2019.04**	(PEA)_2_(MA)_m-1_ PbBr_3m+1_	2D		30.3			20.18	FTO/Buf-HILs/(PEA)_2_(MA)_m-1_PbBr_3m+1_ /TPBi/LiF/Al	[276]
**2019.04**	PMA_2_FA_2_Pb_3_Br_10_	2D	10.2			14,800	43.6	ITO/FPS-TMA/PMA_2_FA_2_Pb_3_Br_10_/TPBi /LiF/Al	[215]
**2019.07**	BA-MAPb (Br/I)_3_	2D/3D	7.42					ITO/Poly-TPD/BA-MAPb(Br/I)_3_ /Bphen/LiQ/Al	[277]
**2020.06**	FAPbBr_3_/CsPbBr_3_ NCs	3D	8.1	93	504	1758		ITO/Poly-TPD/ PeNCs/TPBI/LiQ/Al	[278]
**2021.11**	(DDAxHDA_1−x_)Cs_n−1_PbnBr_3n+1_	Q-2D	12.85	41.5	512	2726		ITO/PEDOT:PSS /(DDA_x_HDA_1-x_)Csn1PbnBr_3n+1_/TPBi/LiF/Al	[279]
**2022.10**	CsPbBr_3_-PEO	3D	12.8			10,737		TO/PEDOT:PSS/PVK-CBP/CsPbBr_3_-PEO /PMMA/AgNWs	[280]
**2023.01**	BMIMBF_4_-CsPbBr_3_	3D	13.75		523	328,000		ITO/PEDOT:PSS/IL-CsPbBr_3_ /PMMA/TPBi/LiF/Al	[281]
**RED**									
**2014.08**	MAPbBr_2_I	3D			630	16.2	0.03	ITO/PEDOT:PSS/CH_3_NH_3_PbBr_3_/F_8_/Ca/Ag	[255]
**2018.02**	FAPbI_3_	3D	12.7					ITO/PEIE-ZnO/perovskite (30 nm)/TFB/MoO_3_ /Au	[223]
**2018.10**	FAPbI_3_	3D	20.7	70				ITO/ZnO-PEIE/Organic layer/FAPbI_3_ /TFBMoO_x_/Au	[222]
**2018.11**	MAPbI_3_	3D	13.5					ITO/MAPbI_3_/LiF/Al	[282]
**2018.11**	MAPbI_3_	3D	14.3		755			ITO/Poly-TPD/MAPbI_3_/TPBi/Al	[273]
**2018.11**	FAPbI_3_	3D	10.1		771			ITO/Poly-TPD/FAPbI_3_/TPBi/Al	[273]
**2018.11**	TFB-PFO	2D/3D	20.1		800			ITO/MZO/PEIE/PPBH/TFB-PFO/MoO_x_/Au	[283]
**2019.01**	MAPbI_3_	QDs	15		750			ITO/Poly-TPD/MAPbI_3_/TPBi/LiF/Al	[284]
**2019.03**	FAPbI_3_	3D	21.6					ITO/ZnO:PEIE/FAPbI_3_/TFB/MoO_x_/Au	[285]
**2019.07**	EDBE-FA_3_Pb_4_I_13_	2D	12		803			ITO/ZnMgO/EDBE/EDBEFA_3_Pb_4_I_13_/TFB/MoO_3_/Au	[9]
**2021.03**	MAPb(I_1−x_Br_x_)_3_	3D	20.3		620			TO/PEDOT:PSS/Poly-TPD/MAPb(I_1−x_Br_x_)_3_/TPBi/LiF/Al	[286]
**2022.02**	CF_3_PEAI-CsPbI_3_	QDs	12.5		685	4550		ITO/ZnO/PEI/CF_3_PEAI- CsPbI_3_/TCTA/MnO_2_	[287]
**2022.06**	EDABr_2_	2D	17.03		671	10,745		ITO/ZnO/PEIE/EDABr_2_/TPBi/LiF/Au	[288]
**2023.08**	PPT	Q-2D	26.2		730			ITO/Poly-TPD/PVP/PPT/TPBi/LiF/Al	[289]
**2023.09**	PEA_2_CsPb_2_I_7_	Q-2D	20.73		656	6483		ITO/PEDOT:PSS/PTAA/PVK/PEA_2_CsPb_2_I_7_/MoO_3_/Ag	[290]

### 3.3. Single-Layer Halide Perovskite Light Emitting

The single-layer halide perovskite light emitting devices have only a metal electrode, ITO, and a halide perovskite light emitting layer. There are no obvious PN junctions. Thus, light emitting devices may be a better term here than LED. They have the advantages of simple preparation process, fewer steps, and low cost compared with the multi-layer LEDs.

**Figure 24:**
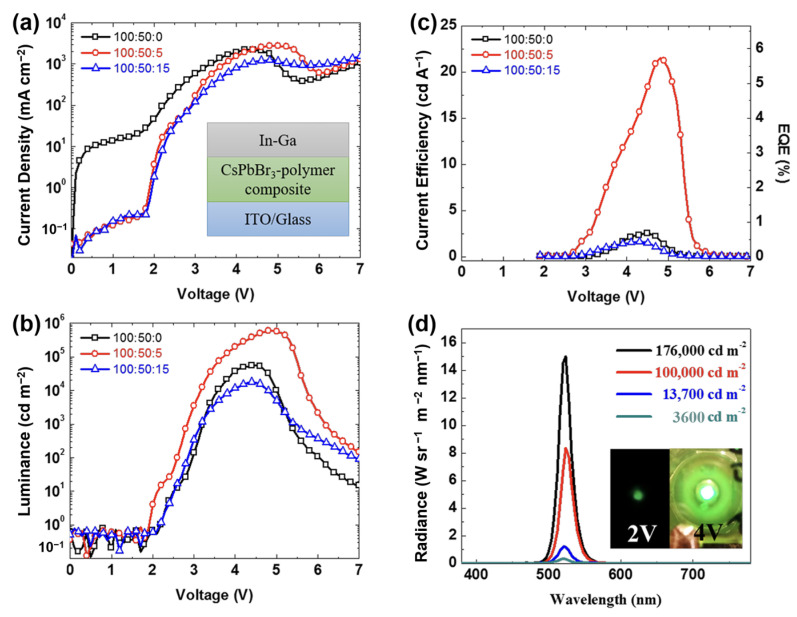
(**a**) Current density and (**b**) luminance vs. voltage characteristics of the single-layer LEDs with different PVP compositions in the emissive layers. The inset in panel (**a**) shows the LED device structure as “ITO anode/CsPbBr_3_-polymer composite/In-Ga cathode”. (**c**) Current efficiency/EQE versus voltage characteristics of the single-layer LEDs. (**d**) Electroluminescence spectra collected at various luminance intensities from a device with CsPbBr_3_:PEO:PVP = 100:50:5 in the emissive layer. The insets are photos of lit devices operating at 2 V bias in the dark (left) and at 4 V bias at an indoor lighting environment (right) [210].

In 2015, Li prepared single-layer halide perovskite light emitting devices made of halide perovskites and poly(ethylene oxide) composite films [260]. The halide perovskite layer was spin-coated between indium tin oxide and indium-gallium alloy. The single-layer light emitting device exhibited a low on-voltage and high brightness due to ionic conductivity of the composite film and the p-i junction was formed. Bade used ITO or carbon nanotubes (CNTs) as an anode, and a printed composite film oxide (PEO) composed of methylammonium bromide (Br-Pero) and poly(ethylene) as a light emitting layer [263]. Silver nanowires used as cathodes and the manufacturing can be carried out in air. The device on ITO/glass had a low on-voltage of 2.6 V, a maximum brightness intensity of 21,014 cd/m^2^, and a maximum external quantum efficiency (EQE) of 1.1%, and the device on the CNT/polymer can be strained to a radius of curvature of 5 mm. Mirershadi synthesized a tunable band gap of CH_3_NH_3_PbX_3_ (X=Br, Cl) and produced a halide perovskite-based monolayer and halide perovskite-based double layer device [291]. Using electron beam deposition techniques, CH_3_NH_3_PbX_3_ was deposited on ITO to form a thin film. Vassilakopoulou synthesized a mixture of quasi-two-dimensional hydrophobic halide perovskite semiconductors, which were spin-coated on ITO to produce single-layer light emitting devices [292]. It was found that a mixture of 3D halide perovskites and unprotonated amines provides a near-semiconductor property. It can also be adjusted by simple halide substitution, and can exhibit a strong bound exciton state, and the oscillator strength increases at room temperature. Light emitting device fabrication was achieved by a single deposition of the hydrophobic mixture, reducing device complexity, cost, and degradability. Li (Figure 24) et al. prepared a single-layer light emitting device by spin- coating CsPbBr_3_ onto ITO using an In-Ga alloy without the EIL or HIL [210]. The light emitting layer exhibited a sub-band gap conduction voltage. The device had 591,197 cd/m^2^ luminance at 4.8 V with 5.7% external quantum efficiency and 14.1 lm/W power efficiency. These researches show that the high electron and hole injection efficiencies can be achieved in halide perovskite light emitting devices without EIL or HIL, which can greatly reduce the cost of halide perovskite light emitting devices.

### 3.4. Challenges and Future

Halide perovskite light emitting devices have shown great potential so far, not only with full bandgap, but also with high brightness, high external quantum efficiency, and a wider color gamut. However, there are still some problems, most notably the stability of the device and the efficiency of the device.

The instability of the device is mainly reflected in the instability of the material itself and the instability of the interface when the device is formed. To solve this problem, we can establish the Ruddlesden–Popper phase [276], using ions and barriers to inhibit ion migration [221,240,277,285], to prepare a uniform bulk polycrystalline halide perovskite layer [293], and to produce stable materials. Meanwhile, the use of the multifunctional molecular additive can slow down the crystallization rate of halide perovskites, promote the formation of high-quality and large-grain perovskite films, and form coordination bonds with Pb^2+^ to passivate uncoordinated Pb^2+^ defects, thereby it can improve the stability of the films [294]. The following strategies were also used: titanium ore nanoparticles [223,246], the preparation of core-shell structure [239], the setting of A-site ions, etc. [295,296].

Reducing contact between halide perovskites, air and water naturally enhances its stability in these environments. A straightforward approach is to embed the halide perovskite within silica spheres. This method effectively minimizes direct exposure to potentially degrading elements, thereby improving the halide perovskite materials’ durability and longevity in various conditions [297]. Three-dimensional halide perovskites are prone to surface defects, leading to significant Shockley–Read–Hall (SRH) recombination and insufficient interaction between components, resulting in lower efficiency and stability. In contrast, two-dimensional (2D) halide perovskites exhibit superior stability in humid and thermal environments. This distinction highlights the potential of 2D halide perovskites for more durable and efficient photovoltaic applications compared to their 3D counterparts. Therefore, another strategy is to surface-passivate 3D halide perovskites with 2D halide perovskites, thereby obtaining mixed halide perovskites (2D/3D) that exhibit better stability without compromising efficiency. This approach leverages the inherent stability of 2D structures to enhance the overall performance and durability of halide perovskite-based devices [298].

The toxicity of halide perovskites poses a significant challenge to their widespread application. Lead free halide perovskites are considered potential substitutes due to their non-toxicity and high stability. A recent method to stabilize lead free halide perovskite materials involves substituting Pb^2+^ with heterovalent M^3+^ cations. A promising candidate for this type of substitution is the non-toxic Bi^3+^, which is isoelectronic with Pb^2+^. This approach aims to mitigate the environmental and health concerns associated with halide perovskites while maintaining their desirable optoelectronic properties [299].

To obtain a more efficient device, we can also reduce the non-radiative recombination from the material itself and reduce the loss caused by the transport layer interface. More uniform halide perovskite films can be created [233,244], such as optimized crystals [271,296], optimized film thickness [272], better contact transport layers [216,300,301], reduced trap defects [35,302], A-site ion setting, and other methods [221].

## 4. Conclusions

In this review, the recent significant advances in the field of halide perovskite materials for solar cells and light emitting are highlighted.

We summarized the application of halide perovskite materials in the research and application in:

1. Halide perovskite solar cells: due to the suitable energy gap of the perovskite material, high absorption coefficient, low electron–hole pair binding energy, balanced carrier mobility, long photon carrier lifetime, etc. These advantages make it as the most potential in solar cell materials. However, halide perovskite solar cells still have some problems in stability, which are susceptible to temperature, moisture, oxygen, and other conditions, and the stability of the hole layer of solar cells is also a direction that we need to explore in depth.
2. Light emitting devices: materials in semiconductor light emitting devices (including organic LEDs) typically need to be processed at high temperatures in a vacuum chamber to ensure that the resulting semiconductor is pure. However, perovskites can be prepared by the simple wet chemistry method. And light emitting devices based on halide perovskite materials have the advantage that the band gap is adjustable. However, the most critical issue for light emitting is the stability problem, as well as the toxicity of halide perovskite. It is also an important research direction to produce high-efficiency Pb free halide perovskite light emitting devices.

Although halide perovskite materials have excellent performance in both solar cells and light emitting, there are still many problems to be solved at present, the most important of which are the stability problems of halide perovskite and HTM and lead pollution. The preparation of high-stability, high-efficiency greener halide perovskite materials is the most important direction in the future. With the help of AI/machine learning, these problems will be solved [303,304].

## Figures and Tables

**Table 1:** Methods to change the crystallinity of halide perovskite materials.

Methods	Key Conditions	η, *V_oc_*, *J_sc_*, FF	Ref.
Annealing, pl	1 step, 90 °C, 450–500 nm pkt	11.4, 0.89, 20.3, 0.64	[133]
Annealing, pl	2 steps, 150 °C, 60 min	12, 0.96, 18.05, 0.69	[129]
MAI conc. po	2 steps spin, cubic	17, 1.06, 21.6, 0.74	[128]
Substr. temp. po	1 step, 80 °C, 150 °C, 45 min	5.4, 1.24, 7.8, 0.56	[127]
Annealing, po, pl	1 step, 130 °C, short, fast	13.5, 0.94, 21.5, 0.69	[130]
Moisture, pl	1 step, ann. = 90 °C, hum. = 35%	17.1, 1.05, 20.3, 0.80	[14]
Solvent, pl	1 step, 20%wt, DMF: r-BL = 97:3 (*v*/*v*)	8.84, 0.92, 8.74, 0.76	[131]
Solvent, pl	2 steps, DMSO 100 °C, 1 h, 650 nm pkt	15.6, 0.96, 21.0, 0.76	[134]
Solvent, po	2 steps, DMSO, Toluene	16.4, 1.1, 19.58, 0.76	[135]
Gas/solid	2 steps, HTM free	10.6, 0.82, 18.3, 0.71	[136]
Annealing	1 step, 150 °C, short, fast	21.4, 1.14, 23.2, 0.797	[137]
Solvent	2 steps, DMF/DMSO = 4:1, toluene	20.1, 1.114, 23.34, 77.31	[138]
Solvent	1 step, 100 °C, 90 min, toluene	18.9, 1.06, 22.65, 76.3	[139]
Solvent	1 step, DMF/DMSO = 4:1	18.5, 1.07, 23.6, 74.9	[140]
Annealing	1 step, DMF/DMSO = 4:1, 100 °C, 1 h	17.46, 1.073, 22.41, 0.726	[106]
Solvent	1 step, DMF/DMSO = 4:1, 100 °C, 80 min chlorobenzene	21.4, 1.169, 23.91, 76.5	[141]
Solvent	2 steps, 100 °C, 5 min, isopropanol	14.6, 0.98, 21.9, 0.685	[142]
Solvent	2 steps, 65 °C, 2 min, 100 °C, 5 min	20.4, 1.1, 23.6, 0.79	[143]
Annealing	1 step, DMF/DMSO = 4:1, 130 °C, 60 min	20.93, 1.16, 23.65, 0.763	[144]
Annealing	1 step, 100 °C, 90 min	17.2, 1.1, 20.3, 0.761	[145]
Annealing	2 steps, 100 °C, 1 h	17.53, 1.09, 20.81, 77.51	[102]
Annealing	2 steps, 100 °C, 10 min	20.9, 1.15, 23.22, 77.62	[107]
Annealing	1 step, 105 °C, 10 min	21.6, 1.18, 22.5, 0.83	[105]

**Table 2:** Summary of the important progress of efficiency (*η*) records for halide perovskite solar cells in the past 15 years.

Year	Event	Others	Ref.
**2009.04**	1st cell	η = 3.8%	[6]
**2012.11**	Al_2_O_3_, over 10%	η = 10.9%	[95]
**2013.02**	CNPB, PDI	*V_oc_* = 1.3 V	[146]
**2013.05**	Rutile TiO_2_, NW rod	η = 9.4%	[97]
**2013.07**	Eff. Over 15%	2 steps	[91]
	ZrO_2_	η = 10.8%	[147]
**2013.09**	Vapor deposition	η = 15.4%	[71]
**2013.10**	Over 1 µm charge diffusion	Abs. Coe. = 57 k/cm	[41]
**2013.12**	HTM:CuI	stable than spiro	[118]
	Graphene	η = 15.6%	[148]
**2014.01**	Flexible, low temperature	η = 11.5%	[149]
	NH_2_CH = NH_2_PbI_3_	η = 7.5%	[119]
**2014.02**	HTM free	η = 10.5%	[122]
	Additive	η = 11.8%	[62]
	ZnO	η = 4.8%	[99]
	Graphene QD	η = 10.2%	[150]
**2014.03**	TiCl_4_, low temp., rutile	η = 13.7%	[98]
**2014.05**	Pb free, SnI_2_	η = 5.73%	[124]
	HTM:CuSCN	η = 12.4%	[119]
**2014.08**	Y:TiO_2_	η = 19.4%	[96]
**2014.12**	TiO_2_ NWs, rutile	η = 11.7%	[89]
**2015.01**	175 μm diffusion length	1 cm single crystal	[59]
**2015.02**	ZnO + 3-aminopropanoic acid	η = 15.67%	[151]
**2015.03**	TiO_2_ + ZrO_2_ + NiO + C	η = 14.9%, no spiro	[152]
**2015.05**	FAPbI_3_	η = 20.2%	[86]
	CuSCN, Inverted Planar, C_60_	η = 16.6%	[153]
**2017.06**	Iodide management	η = 22.1%	[154]
**2017.10**	CsPbI_3_ quantum dot	η = 13.43%	[155]
**2017.11**	HTM:CuSCN	η = 20.4%	[120]
**2017.12**	HTM:(Ta-WO_x_)/conjugated polymer	η = 21.2%	[156]
**2018.07**	The highest efficiency of F-PSCs	η = 22.7%	[157]
	No MA and all inorganic	η = 20.35%	[158]
	The highest efficiency of 2D PSC	η = 16.92%	[159]
	Laminated battery:Cu(In,Ga)Se_2_	η = 22.43%	[160]
	CsPbI_2_Br high efficiency	η = 14.78%	[161]
**2019.01**	Eu^3+^-Eu^2+^ ion redox	η = 21.52%	[162]
**2019.03**	HTM: poly(3-hexylthiophene)	η = 22.7%	[8]
**2019.04**	Reach the Shockley-Queisse limit	*V_oc_* = 1.18 V	[163]
	Open circuit voltage record	*V_oc_* = 1.31 V	[164]
	Highest efficiency of Rutile TiO_2_ Electron Transport Layer	η = 20.9%	[107]
**2019.05**	The highest efficiency of all inorganic perovskite	η = 22.6%	[165]
**2019.07**	Ionic liquid additives	long-term stability	[166]
**2020.04**	Narrow-bandgap mixed lead-tin	η = 24.2%	[167]
**2020.09**	p-n junction and in chemical-type	metallization	[168]
**2021.01**	CsPbI_3_	η = 20.37%	[169]
**2021.03**	Bismuth iodide interfacial layer	η = 24.07%	[170]
**2021.06**	(FAPbI3)_0_._85_(MAPbBr3)_0_._15_	η = 25.8%	[171]
**2021.08**	Coupling Cl-bonded SnO_2_	η = 22.6%	[172]
**2022.08**	NIR polymer DTBTI-based BHJ	η = 24.27%	[173]
**2022.12**	4-Terminal inorganic perovskite/organic tandem	η = 22.34%	[174]
**2023.11**	1-(phenylsulfonyl)pyrrole	η = 26.1%	[7]

**Table 3:** Halide perovskite solar cells and the stability.

Year	Method	η/Time (h)/Efficiency Residue (%)	Ref.
**Pero.**			
**2016.04**	Scraper coating/copper as cathode	18.3/720/~90	[175]
**2016.09**	Introduction of phenethyl ammonium iodide (PEAI)	17.7/384/90	[176]
**2017.02**	K-doped CsPbI_2_Br	10/120/80	[177]
**2017.02**	Isomers—pure double PCBM assist	19.9/600/96	[178]
**2017.05**	Add thiosemicarbazone	19.19/500/80	[179]
**2017.06**	2D/3D multidimensional interface	14.6/10,000/~75	[180]
**2017.08**	2D-3D heterojunction	17.5/1000/80	[181]
**2018.01**	SnO_2_/FAMACs/EH44/MoO_x_/Al architecture	22.7/1000/94	[182]
**2019.03**	HTAB treatment	22.7/1370/95	[8]
**2019.05**	Low poly-SiO_2_ in situ coated	21.5/5200/80	[183]
**2019.05**	In situ passivation of phenethyl iodide	23.32/500/80	[163]
**2019.06**	Linear alkyl ammonium bromide treatment	22.6/ (Wide band gap)	[165]
**2019.08**	PbSO_4_, Pb_3_(PO_4_)_2_ in situ passivation	22.1/1200/96.8	[184]
**2021.08**	Coupling Cl-bonded SnO_2_	25.8/500/90	[172]
**2022.03**	Ionic liquid butylammonium acetate	20.1/700/79.5	[185]
**2022.11**	Precursor engineering	21.26/300/90	[186]
**2023.06**	4,4′-cyclohexylbis[N,N-bis(4-methylphenyl)aniline]	23.15/720/90	[187]
**HTM**			
**2016.01**	Crosslinkable silane molecules bonded to fullerenes	19.5/720/90	[188]
**2016.12**	Using CuGaO_2_	18.5/720/83	[189]
**2017.11**	Use copper thiocyanate	20/1000/95	[120]
**2018.07**	End cap screw-OMeTAD	22.6/500/95	[117]
**2019.07**	Spiro-OMeTAD layer using MoS_2_	20.18/300/85	[190]
**2022.09**	Solution Processed Ternary Tin (II) Alloy	23.2/1500/85	[191]
**2022.10**	Use PFBTI as the HTM deliver	22.2/500/80	[192]
**2023.06**	2% Cu@ZnCo_2_O_4_	15.79/1800/90	[193]
**ETL**			
**2016.12**	Doped n-type fullerene layer	16/3400/66	[194]
**2017.02**	Chlorinated TiO_2_	20.1/500/97.5	[195]
**2021.11**	Infrared radiation annealing	22/1008/92	[196]
**2022.09**	TAC-doped SnO_2_	21.58/1000/88	[197]
**2023.04**	pre-buried 3-aminopropionic acid hydroiodide	23.36/720/92	[198]
